# Depicting the cellular complexity of pancreatic adenocarcinoma by Imaging Mass Cytometry: focus on cancer-associated fibroblasts

**DOI:** 10.3389/fimmu.2024.1472433

**Published:** 2024-11-07

**Authors:** Marco Erreni, Maria Rita Fumagalli, Raffaella D’Anna, Mauro Sollai, Silvia Bozzarelli, Gennaro Nappo, Damiano Zanini, Raffaella Parente, Cecilia Garlanda, Lorenza Rimassa, Luigi Maria Terracciano, Subhra K. Biswas, Alessandro Zerbi, Alberto Mantovani, Andrea Doni

**Affiliations:** ^1^ Unit of Multiscale and Nanostructural Imaging, IRCCS Humanitas Research Hospital, Milan, Italy; ^2^ Department of Biomedical Sciences, Humanitas University, Milan, Italy; ^3^ Pathology Unit, IRCCS Humanitas Research Hospital, Milan, Italy; ^4^ Medical Oncology and Hematology Unit, Humanitas Cancer Center, IRCCS Humanitas Research Hospital, Milan, Italy; ^5^ Pancreatic Surgery Unit, IRCCS Humanitas Research Hospital, Milan, Italy; ^6^ IRCCS Humanitas Research Hospital, Milan, Italy; ^7^ Singapore Immunology Network (SIgN), Agency for Science, Technology and Research (A*STAR), Singapore, Singapore; ^8^ William Harvey Research Institute, Queen Mary University of London, London, United Kingdom

**Keywords:** multiplexed histopathology, Imaging Mass Cytometry, pancreatic cancer, tumor microenvironment, cancer-associated fibroblasts (CAFs)

## Abstract

**Introduction:**

Pancreatic ductal adenocarcinoma (PDAC) represents the complexity of interaction between cancer and cells of the tumor microenvironment (TME). Immune cells affect tumor cell behavior, thus driving cancer progression. Cancer-associated fibroblasts (CAFs) are responsible of the desmoplastic and fibrotic reaction by regulating deposition and remodeling of extracellular matrix (ECM). As tumor-promoting cells abundant in PDAC ECM, CAFs represent promising targets for novel anticancer interventions. However, relevant clinical trials are hampered by the lack of specific markers and elusive differences among CAF subtypes. Indeed, while single-cell transcriptomic analyses have provided important information on the cellular constituents of PDACs and related molecular pathways, studies based on the identification of protein markers in tissues aimed at identifying CAF subtypes and new molecular targets result incomplete.

**Methods:**

Herein, we applied multiplexed Imaging Mass Cytometry (IMC) at single-cell resolution on 8 human PDAC tissues to depict the PDAC composing cells, and profiling immune cells, endothelial cells (ECs), as well as endocrine cells and tumor cells.

**Results:**

We focused on CAFs by characterizing up to 19 clusters distinguished by phenotype, spatiality, and interaction with immune and tumor cells. We report evidence that specific subtypes of CAFs (CAFs 10 and 11) predominantly are enriched at the tumor-stroma interface and closely associated with tumor cells. CAFs expressing different combinations of FAP, podoplanin and cadherin-11, were associated with a higher level of CA19-9. Moreover, we identified specific subsets of FAP^+^ and podoplanin^+^/cadherin-11^+^ CAFs enriched in patients with negative prognosis.

**Discussion:**

The present study provides new general insights into the complexity of the PDAC microenvironment by defining phenotypic heterogeneities and spatial distributions of CAFs, thus suggesting different functions of their subtypes in the PDAC microenvironment.

## 1 Introduction

Pancreatic ductal adenocarcinoma (PDAC) is one of the most lethal types of cancer, with a 5-years survival rate of 11% only (1). This poor prognosis is mainly due to the inability to detect the disease until late, often metastatic, tumor stage (2). Further, diagnosis is complicated by the asymptomatic evolution of the disease, the lack of diagnostic biomarkers, the absence of attributable risk factors for the majority of patients and the difficult-to-access anatomical location of the pancreas, which limits the routine screening intervention (3, 4). Although 10-15% of cases can be ascribed to germline mutations or known risk factors, the majority of PDAC develops as a consequence of accumulating mutations in several genes, including *KRAS*, *p53*, *SMAD4* and *CDKN2A*, which results in the formation of pre-cancerous lesions, such as Pancreatic Intraepithelial Neoplasia (PanIN) and Intrapapillary Mucinous Neoplasia (IPMN), that possibly evolve to invasive cancer (5–8). Beside mutations that drive the neoplastic morphological alterations of pancreatic epithelial cells, PDAC is characterized by a massive infiltration of activated cancer-associated fibroblasts (CAFs), responsible for the deposition of extracellular matrix components and leading to a desmoplastic reaction, that shapes a tumor microenvironment (TME) composed by a dense stroma, a leaky vascular system and suppressive immune cell populations (9–11). The resulting TME, which can develop up to the 90% of the entire tumor mass, is indeed the main responsible of the heterogeneity, aggressive biology and resistance to therapy of the disease (9, 12). Although the limit in the 5-years survival rate, PDAC survival statistics have doubled over the past decades, due to the improved therapeutic approaches and clinical care (13).

Cell heterogeneity in PDAC has been widely investigated using single-cells transcriptomic approaches, but only few studies analyzed the protein expressed by the different cell subpopulations that compose the PDAC microenvironment (14–16). In the last decade, multiplexed Imaging Mass Cytometry (IMC) has emerged as a powerful technology to dissect the cell landscape of several TME (17–19). IMC combines conventional histology with mass cytometry to identify up to 35-40 metal-tagged antibodies, avoiding limitations related to fluorescence-based imaging technologies, including autofluorescence and spectral overlap (17, 20). Although several studies used conventional immunohistochemistry or fluorescence-based imaging to target markers of PDAC microenvironment (15, 16, 21, 22), fewer are the investigations conducted using the multiplexed IMC technology (23, 24). In addition, these studies mainly focused on the immune cell composition in the PDAC microenvironment, with a limited analysis of the CAF phenotype and localization.

In this manuscript, we applied a 31-antibody panel to define the organization and composition of the PDAC tumor microenvironment by IMC. We focused on the phenotype and the spatial localization of different CAF subpopulations, together with their relationship with immune, endothelial cells (ECs) and tumor cells. With this approach, we provide a comprehensive analysis of the PDAC microenvironment with the aim of better defining its cellular complexity, thereby identifying subtypes and cell signatures of relevance and useful in diagnosis and instrumental for new treatment strategies.

## 2 Materials and methods

### 2.1 Human samples and study design

The analyzed cohort includes 8 patients diagnosed with PDAC surgically resected at the Humanitas Research Hospital between 2022 and 2023. Patients’ histopathological and clinical features are listed in **Supplementary Table 1**. Patients had not received any therapy before resection. Written informed consent was obtained for each patient included in the study. The study protocol was in accordance with ethical guidelines established in the 1975 Declaration of Helsinki and was approved by the local ethical committee (Authorization n° 3801).

### 2.2 Histopathological evaluation

5μm-thick formalin-fixed, paraffin-embedded (FFPE) sections from PDAC tissue blocks were deparaffinized in xylen and rehydrated through a graded alcohol series. Tissue sections were stained with Hematoxylin (Histo-Line Laboratories, Pantigliate (MI) - Italy) for 15 minutes, extensively washed in H_2_O for 10 minutes, and then stained with Eosin (Histo-Line Laboratories, Pantigliate (MI) - Italy) for 7 minutes. After a rapid wash in H_2_O, slides were dehydrated through a graded alcohol series, washed in xylene and then mounted with Eukitt (Sigma-Aldrich, St. Louis, Missouri, USA). Whole-slide scans were acquired by a ZEISS Axio Scan Z1 Slide Scanner and visualized with QuPath software (version 0.5.1).

### 2.3 Tissue staining

2μm-thick FFPE sections from PDAC tissue blocks were deparaffinized in xylen and rehydrated through a graded alcohol series. Slides were then incubated with EDTA, pH 9 antigen retrieval solution (Agilent Technologies, Santa Clara, CA 95051, USA) in a water bath at 98°C for 20 minutes, followed by a 10-minutes cooling down in antigen retrieval solution and by an additional 10-minutes cooling down in distilled water. To prevent non-specific antibody binding, slides were incubated in PBS supplemented with Ca^2+^ and Mg^2+^ (PBS^2+^) (Lonza, Basel, Switzerland) supplemented with 0.1% Triton X-100, 3% BSA (Sigma-Aldrich, St. Louis, Missouri, USA), 5% Normal Mouse (Biosera, Cholet, France)/Rat(Sigma-Aldrich, St. Louis, Missouri, USA)/Rabbit(Dako, Agilent Technologies, Santa Clara, CA 95051, USA)/Goat(Sigma-Aldrich, St. Louis, Missouri, USA)/Sheep (Sigma-Aldrich, St. Louis, Missouri, USA) serum, for 45 minutes at room temperature in a humified chamber. Slides were then incubated with the metal-conjugated antibody mix, diluted in PBS^2+^ supplemented with 0.01% Triton X-100, 0.3% BSA (Sigma-Aldrich, St. Louis, Missouri, USA), 0.5% Normal Mouse (Biosera, Cholet, France)/Rat(Sigma-Aldrich, St. Louis, Missouri, USA)/Rabbit(Dako, Agilent Technologies, Santa Clara, CA 95051, USA)/Goat(Sigma-Aldrich, St. Louis, Missouri, USA), Sheep (Sigma-Aldrich, St. Louis, Missouri, USA) Serum, overnight at 4°C in a humified chamber. Slides were washed 4 times, 5 minutes each, in PBS^2+^ 0.05% Tween–20 (Merck, Darmstadt, Germany). For nuclear staining, tissues were then incubated with 0.3 µM Ir191/193 (Standard Biotools, South San Francisco, CA, USA) in PBS^2+^ for 30 minutes at room temperature. After incubation, tissue sections were washed 3 times, 3 minutes each, in PBS^2+^ 0.05% Tween-20. Finally, sections were washed for 30 seconds in ultrapure H_2_O to remove salt leftovers and quickly airdried. The list of 31 metal-conjugated antibodies used in this study is reported in **Supplementary Table 2**. Metal-tagged antibodies recognizing alpha smooth muscle actin (αSMA), CD163, CD20, CD66b and collagen-I were purchased from Standard Biotools. The remaining antibodies were conjugated to lanthanide isotypes using the Maxpar^®^ X8 Antibody Labelling Kit (Standard Biotools, South San Francisco, CA, USA) according to the manufacturer’s instructions and resuspended in PBS^2+^ and 0.05% NaN_3_. Titration tests were performed for each metal-conjugated antibody to optimize the staining protocol.

### 2.4 IMC data acquisition

Images were acquired with a Hyperion Imaging System (Standard Biotools, South San Francisco, CA, USA). To ensure system stability, the Hyperion Imaging System was routinely calibrated following the manufacturer’s instructions. For each patient, 2 consecutive sections were cut and stained for H&E and IMC staining, respectively, as previously described. On the H&E-dedicated slides, 3 to 5 regions of interest (ROIs), corresponding to tumor regions, were selected by a specialized pathologist. The same regions were then identified on the IMC dedicated slides and 1 mm^2^ ROIs were ablated with a UV laser, with a frequency of 200Hz, at a resolution of approximately 1µm^2^. IMC acquired regions were then revised by a specialized pathologist to confirm the presence of the neoplastic tissue. Antibodies that showed high level of background signal in tumor tissue or did not exhibit a clear staining pattern were excluded from the analysis, resulting in a final panel of 31 metal-tagged antibodies.

### 2.5 Data analysis

IMC image analysis was performed using a custom pipeline as previously described (25). Briefly, hot pixel removal (radius=2, threshold=50) was performed on single–channel images extracted from mcd files. For each channel, low–intensity thresholds were manually settled based on visual inspection and a cutoff was set to at the top 99.99% percentile of expression (or at least at an intensity value of 10 dual counts) calculated over all the considered ROIs. Gaussian filter (r=2) was applied exclusively to estimate of pan-Cytokeratin+ (Pan-Ck^+^), CD45^+^, CD31^+^ and fibroblast activation protein (FAP^+^) positive area to avoid bias due to missing nuclear signal and small debrids.

Tiffs substacks containing the complete list of channels relevant for segmentation and cell classification were created. Ilastik (v1.3.3post3) (26) and CellProfiler (v4.2.1) (27) were used to perform single–pixel classification and cell segmentation. R EBImage package (v4.36) (28) was used to obtain channel intensity and shape parameters for each cell. Objects with area < 10µm^2^, area larger than 1000µm^2^, mean intensity higher than 2 in more than 15 markers, and lower than 0.01 in the markers used for Uniform Manifold Approximation and Projection (UMAP) analysis were discarded. No more than 2% of the cells were discarded based on these criteria.

Following inverse hyperbolic sine transformation and normalization of the data between 1% and 99% of the overall signal, UMAP (https://CRAN.R-project.org/package=umap, v0.2.8) and PhenoGraph (v 0.99) (k=60) algorithms were used for dimensional reduction and clustering analysis. Clusters were assigned to five different cellular populations (tumor, immune, ECs, CAFs, pancreatic islets). PhenoGraph analysis (k=20) was then performed on the five subsets separately, in order to identify clusters of cells misannotated. After reassignment of all the cells to the correct populations, cells were re-clustered (k=20) and annotated into more specific subpopulations, as described in the Results section.

Neighboring cells were identified as those located within 30μm from cells borders using 3D interaction Fiji plugin (mcib3D v4.1.5). Interaction counts score were determined using patch method (p=1) from the imcRtools package (v1.9.0, permutation test n=5000). Patch method gives for a reference subpopulation, and each ROI, the fraction of its cells that have at least one neighbor in the target subpopulation. Cells subpopulations with less than 10 cells for ROI were not included in the statistics. For each pair of cell subtypes, permutation test over their positions allows to obtain an estimate of p-value associated to the observed number of interactions compared to those expected by chance. Interactions were tested separately for each ROI, and considered significant when p-value < 0.01. A score +1 or -1 was associated to each significantly positive or negative (more or less associated than expected by random model) interaction. Non-significant interactions were given score zero. The resulting scores, averaged over all the considered ROIs, were represented in heatmaps. For CAF subtypes, the minimum distance from each cell and tumor cells was evaluated with Cdist function (Rdist v0.05) using center of mass. For each cell we calculated the abundance of different neighboring cells (identified, as above, in a radius <30μm) subpopulation. We considered 29 classes, taking into account tumors and endothelial cells as aggregated macro populations, while subpopulations of CAFs and immune cells were considered in detail. The abundance normalized vectors were used as input for kmeans clustering (k=10, 1500 iterations, 10 initialization sets). The resulting clusters were manually annotated, based on center coordinates and cell subtype enrichment, to identify neighborhoods regions similar in composition (**Supplementary Table 3**).

Relative enrichment of CAFs subtypes in the groups identified by clinical parameters was defined ad –Log10 of the false discovery rate (fdr) from hypergeometric test for the good prognosis group and +Log10 of fdr for bad prognosis group. Thus, larger positive values indicate enrichment in the good prognosis group, while large negative values are indicative of enrichment in the bad prognosis group. Values of fdr were capped to 10^-10^. Values fdr > 0.001 were set to 1, corresponding to enrichment score 0.

### 2.6 Image processing and statistics

Representative images were prepared using ImageJ (Fiji, version 1.54f) software. Gaussian filter was applied to representative images to increase their quality. GraphPad Prism software (version 9.0.2), dittoSeq (v1.6.0) and ggplot2 (v3.4) R packages were used to prepare graphs and to performed statistical analysis. Two-sample, two sided Kolmogorov-Smirnov (KS) test was used to compare distances distribution. Hypergeometric test was performed based on HypeR package (29). False discovery correction was applied to all p-values and reported as p-adjusted (padj).

## 3 Results

### 3.1 The cellular TME of PDAC

**Figure 1:**
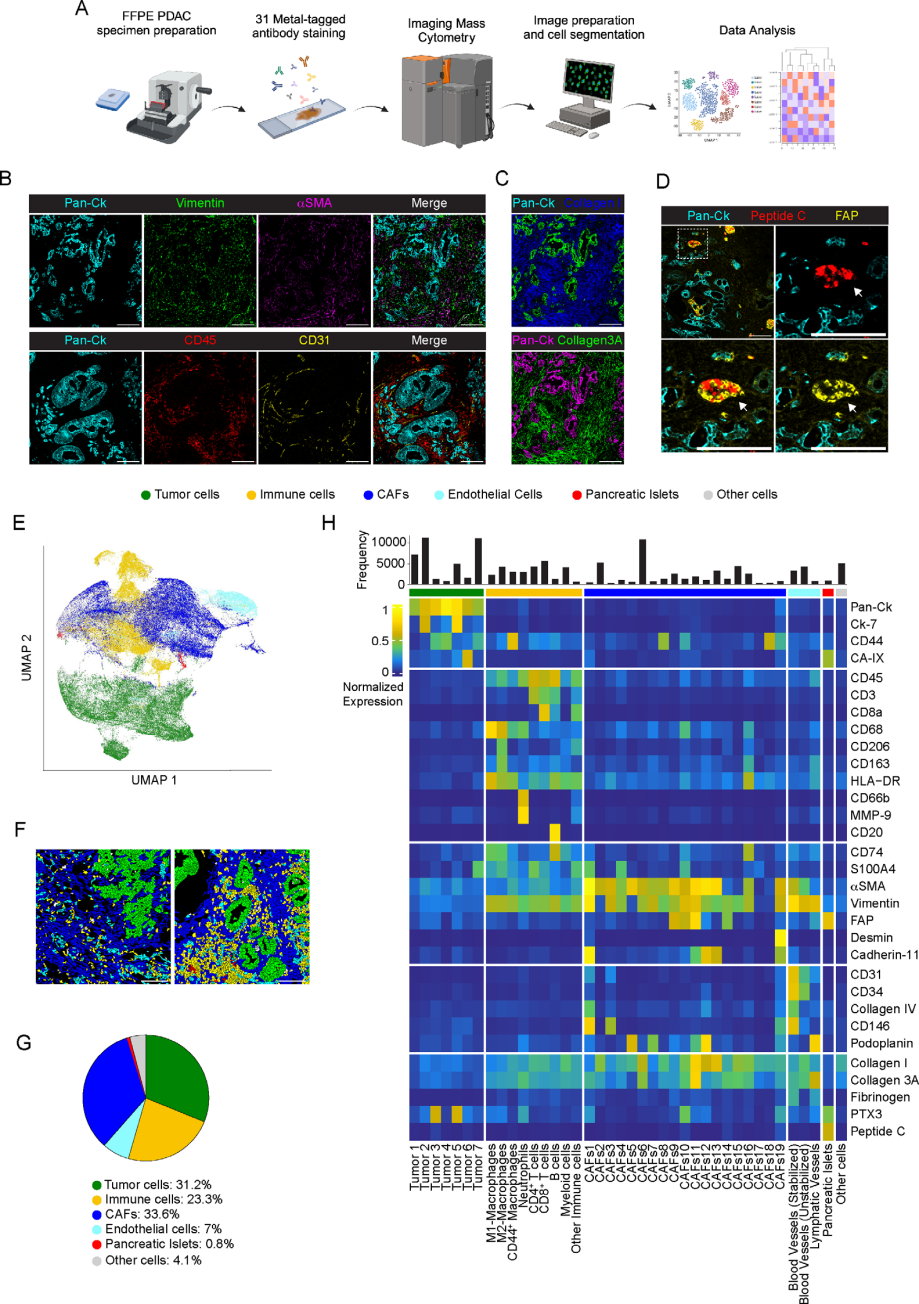
Cellular landscape in PDAC by IMC. **(A)** Schematic view of the IMC analysis workflow. **(B)** Representative images out of 34 acquired ROIs (n=8 PDAC) showing extracted signal contribution of Pan-Ck (cyan), vimentin (green), αSMA (magenta), CD45 (red), CD31 (yellow) and correspondent merged image. Bar: 200µm. **(C)** Upper panel, representative images out of 34 acquired ROIs (n=8 PDAC) showing signal contribution of Pan-Ck (green) and Collagen I (blue). Bar: 200µm. Lower panel, representative images out of 34 acquired ROIs (n=8 PDAC) showing Pan-Ck (magenta) and Collagen 3A (green). Bar: 200µm. **(D)** Representative images out of 34 acquired ROIs (n=8 PDAC) showing Pan-Ck (cyan), peptide C (red) and FAP (yellow). White arrow in the inset indicates the pancreatic islet expressing peptide C (red) and FAP (yellow). Bar: 200µm. **(E)** UMAP representation of PDAC cells annotated into tumor cells, immune cells, CAFs, ECs, pancreatic islet and other cells. **(F)** Representative reconstructed images showing the localization within the PDAC tissues of the segmented cells (black contours) corresponding to tumor cell (green), immune cells (yellow), CAF (blue), ECs (cyan), pancreatic islets (red) and other (gray) cell clusters. **(G)** Frequency of cells belonging to tumor cell (green), immune cell (yellow), CAF (blue), ECs (cyan), pancreatic islets (red) and other cell (gray) clusters, as in the legend. Bar: 200µm. **(H)** Heatmap referring to the normalized expression of each single markers of the acquired images (n=34 ROIs; n=8 PDAC) after PhenoGraph analysis, among the different clusters.

Multiple staining protocols combined with IMC technology at single-cell resolution (**Figure 1A**) were applied to human tissues of PDAC (n=8) (**Supplementary Table 1**). A total of 34 ROIs (1mm^2^ range of n=3-5 per patient) were selected for acquisition, based on histopathological evaluation, including both neoplastic glands from PDAC (with various grades of differentiation) and stromal tissue, with the aim of acquiring regions similarly divided between PDAC and remaining TME (23±11% Pan-Ck^+^ area, n=34 PDAC). As stated in **Supplementary Table 2** and shown in **Supplementary Figure 1A**, tissue sections were stained with 31 metal-tagged antibodies detecting classical markers of tumor cells (Pan-Ck, Ck-7), pancreatic islets (peptide C); monocytic (CD45, CD68), polymorphonuclear (CD66b, MMP-9) and lymphoid (CD45, CD3, CD8, CD20) immune cells; cells composing the blood vessel wall, including vascular ECs (CD31, CD34), smooth muscle cells (CD146) and pericytes (CD146, αSMA); lymphatic ECs (CD31, podoplanin). Detection of differential markers subtyped the cells of mesenchymal origin (vimentin, desmin, cadherin-11, podoplanin, CD74, S100A4, CD44, FAP), which vary according to the functional differentiation and specialization in sites of cancer tissue (30–32). In addition, a definition of the diversity of ECM components (collagen-I, collagen-3A, collagen-IV, fibrinogen, pentraxin 3 (PTX3)) served to assess a functional association with different cell types in the proximity, such as functionality and stability of tumor blood vessels by measuring collagen IV-rich coverage (33). Additional markers included CD206, CD163 and HLA-DR, to reveal a functional state of tumor-associated macrophage (TAM) (M1 versus M2 polarization), whereas evaluation of carbonic anhydrase 9 (CA-IX) expression defined the cancer cell capable in sustaining local acidosis, and hence in favoring cancer progression (34) (**Supplementary Figure 1A**).

In all PDAC tissues analyzed, Pan-Ck^+^ regions are randomly arranged and surrounded by a dense desmoplastic and collagenous stroma enriched of αSMA and vimentin. Dispersed blood vessels (2.1±1.5% CD31^+^ area n=34 ROIs) and immune cells (15.7±8.2% CD45^+^ area; n=34 ROIs) enclosed the tumor cells (**Figures 1B, C**). Isolated epithelial peptide C^+^/FAP^+^ pancreatic islets (35) (0.4±0.6% peptide C^+^ area, range 0-2.3%, 0.8±1.9% FAP^+^ range 0-9%; n=34 ROIs) were distinguished from Pan-Ck^+^ cells (**Figure 1D**).

In a single-cell segmentation analysis, we generated a mask for each ROI of PDAC identifying a total number of 122827 cells (range 12080-18643 cells for n=8 PDAC) (a representative image of single-cell segmentation is shown in **Supplementary Figure 1B**). PhenoGraph analysis generated 32 different clusters, subdivided in tumor cells (Pan-Ck^+^ and Ck-7^+^), immune cells (CD45^+^, CD3^+^, CD68^+^, CD66b^+^, CD20^+^), ECs (CD31^+^, CD34^+^, podoplanin^+^), CAFs (CD45^-^, Pan-Ck^-^, Ck-7^-^, CD31^-^, αSMA^+^, vimentin^+^, CD74^+^, CD44^+^, S100A4^+^, FAP^+^, podoplanin^+^, cadherin-11^+^, desmin^+^), pancreatic islets (peptide C^+^) and stated as other cells for not expressing specific markers. UMAP representation of the annotated cluster is shown in in **Figure 1E**; **Supplementary Figure 2A** and, per single patient, in **Supplementary Figure 2B**. Representative images of the distribution of annotated cell subtypes in PDAC tissue are shown in **Figure 1F**.

CAFs represented the most abundant cell population identified (n=41339 cells, n=8 PDAC; range 21-43.6% per PDAC) and corresponded to 33.6% of the annotated cells, compared with tumor cells (n=38284; 31.2%, n=8; range 22.5-37.2% per PDAC), immune cells (n=28661 cells, 23.3%, n=8; range 12.7-45.5% per PDAC) and ECs (n=8596 cells, 7%, n=8; range 2.9-12.2% per PDAC) (**Figure 1G**). 4.1% of cells (n=5070; n=8; range 0.6-10.4% per PDAC) were not specifically annotated. The clusters identified were homogeneously represented among the different PDAC patients, with a diversity of relative abundance more associated with specific subtypes of CAFs and tumor cells (**Supplementary Figure 2B**).

As shown in the averaged intensity-based heatmap (**Figure 1H**) and in line with transcriptomic studies in PDAC (36), tumor cells were further reclassified into 7 different subtypes, based on the expression of Pan-Ck, Ck-7, CD44, S100A4 and CA-IX. Although PTX3 was recognized as a molecule predominantly associated with cells of mesenchymal origin in PDAC (37), two clusters of tumor cells (Tumor 3, Pan-Ck^+^ PTX3^+^; Tumor 5, Pan-Ck^+^ Ck-7^+^ PTX3^+^) were identified based on its high expression. Immune cells were identified as myeloid cells (CD45^+^, CD68^-^, CD206^+^) and as a whole subtyped in M1-like (CD45^+^, CD68^+^, HLA-DR^+^, CD74^+^), M2-like macrophages (CD45^+^, CD68^+^, CD206^+^, CD163^+^) or CD44-expressing macrophages (CD45^+^, CD68^+^, CD44^+^); neutrophils (CD45^+^, CD66b^+^); CD4^+^ T cells (CD45^+^, CD3^+^, CD8^-^) and CD8^+^ T cells (CD45^+^, CD3^+^, CD8^+^); B cells (CD45^+^, CD20^+^) (**Figure 1H**). On the basis on the selective expression of markers of pericytes (αSMA, CD146) and coverage of a collagen-IV^+^ basement (38), blood vessels were distinguished into functioning and stabilized (CD31^+^, CD34^+^, Collagen-IV^+^, CD146^+^, αSMA^+^, cadherin-11^+^) from non-stabilized (CD31^+^, CD34^+^, collagen-IV^-^, CD146^-^) tumor neo-angiogenesis, as well as in CD31^+^ podoplanin^+^ lymphatic vessels (**Figure 1H**). As reported, CAFs represent a multitude of potentially dynamic and plastic subgroups that change their gene expression profiles based on the stimuli from the environment (39), thereby influencing tumor progression through the tissue fibrotic reaction, the regulation of tissue biomechanical property and the modulation of the immune response to chemotherapy (11). Therefore, detection of multiple CAF markers, including αSMA, vimentin, S100A4, CD74, FAP, desmin, cadherin-11, CD34, CD146, CD44, CA-IX, podoplanin, collagen-I, collagen-3A and PTX3, served to discriminate the subpopulation of CAFs having a different functional impact in PDAC. As shown in **Figure 1H**, CAFs were classified into 19 different clusters.

### 3.2 Profiling of cancer cells

**Figure 2:**
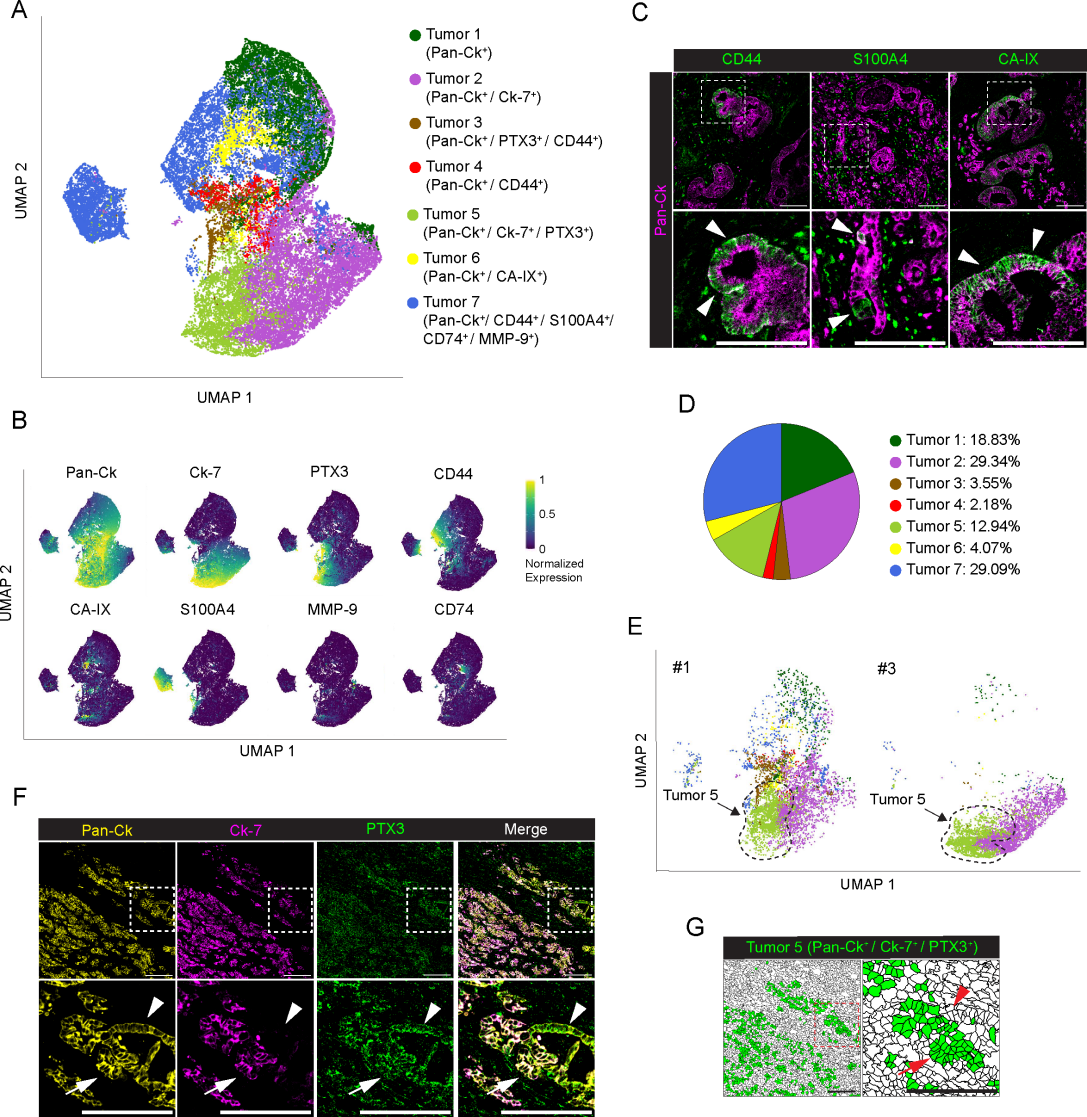
Profiling of tumor cells in PDAC. **(A)** UMAP representation of PDAC tumor cells annotated to 7 tumor cell subtypes, colors as in legend. **(B)** UMAP representation of the normalized expression of single markers in segmented cells annotated as tumor cells. **(C)** Representative images out of 34 acquired ROIs (n=8 PDAC) showing Pan-Ck (magenta) signal in combination with CD44 (green, left panel), S100A4 (green, middle panel) and CA-IX (green, right panel). White arrowheads on the onset (bottom) indicate tumor cells expressing CD44 (left panel), S100A4 (middle panel) and CA-IX (right panel). Bar: 200µm. **(D)** Frequency of cells belonging to the identified tumor subtypes, over the total number of tumor cells, as in legend. **(E)** UMAP representation of the subset of tumor cells in PDAC #1 and #3. Black-dotted line is a guide for the eye. Colors and UMAP coordinates as in **(A)**. **(F)** Representative images of Pan-Ck (yellow), Ck-7 (magenta) and PTX3 (green) signal in the PDAC #1. White arrows and white arrowheads in the inset show tumor cells co-expressing Pan-Ck/Ck-7/PTX3 (Tumor 5) or Pan-Ck/PTX3 alone, respectively. Bar: 200µm. **(G)** Representative images of the same region represented in **(F)**, showing the tissue localization of segmented cells (black contours) annotated as Tumor 5 (green) in PDAC #1. Red arrows and red arrowheads in the inset show tumor cells co-expressing Pan-Ck/Ck-7/PTX3 (Tumor 5) or Pan-Ck/PTX3 alone, respectively. Bar: 200µm.

Tumor cells (n=38284; 31.2%, n=8; range 22.5-37.2% per PDAC) were annotated into 7 different subtypes, based on the expression of Pan-Ck, cytokeratin 7 (Ck-7), CD44, S100A4, PTX3, CA-IX, CD74 (**Figures 2A, B**). Although the expression levels changed among the subtypes, all tumor cells expressed Pan-Ck. A cluster with an exclusive expression of Pan-Ck alone corresponded to 18.8% (n=8; range 1.1-73.5% per PDAC) of tumor cells (Tumor 1), whereas majority of tumor cells expressed both Pan-Ck and Ck-7 (Tumor 2, n=8; range 1.1-74.0% per PDAC). Some clusters expressed a combination of markers associated with tumor proliferation and invasiveness (40), such as CD44, S100A4, CD74 and MMP-9 (Tumor 7, 29.1%, n=8; range 0.7-81.5% per PDAC) (**Figure 2A–C**). In particular, expression of CD44, a classical marker associated with epithelial-to-mesenchymal transition and poor prognosis of PDAC progression and metastasis (41, 42), was found associated with Tumor 3 (3.6%, range 0.2-9.0% per PDAC), Tumor 4 (2.2%, range 0-10.0% per PDAC) and Tumor 7 subtypes (**Figures 2A–C**). Few cells in the Tumor 6 subtype (4.1%, n=8; range 0.2-11.5% per PDAC) expressed the hypoxic marker CA-IX (**Figures 2B, D**). Therefore, as shown by IMC analysis, PDAC consists of a phenotypic diversity (n=7 identified clusters) of tumor cells potentially associated with different capacity for tumorigenesis and metastasis. As shown by UMAP in **Supplementary Figure 3**, for the same clusters, heterogeneity in the expression of the same markers was observed among the PDAC patients analyzed.

PTX3 is a humoral innate immune molecule produced by macrophages (43) and mesenchymal cells (37), which plays a role of extrinsic oncosuppressor of cancer by regulating complement-dependent tumor-promoting inflammation (43). On the other hand, PTX3 was found elevated in PDAC tissue and associated with an increased capacity of cancer cells to invade ECM (37). As observed in other tumors, PTX3 produced by cancer cells promote tumor progression by promoting invasiveness and migration (44, 45). Interestingly, we found that the Tumor 5 (12.9%, range 0-43.7% per PDAC, **Figure 2D**), expressing Pan-Ck, Ck-7 and PTX3 was almost exclusively found in PDAC #1 and #3 only, counting for the 90% of all identified Tumor 5 cells (**Figure 2E**), who were diagnosed with distant metastasis at the time of surgery (**Supplementary Table 1**). Co-expression of Pan-Ck, Ck-7 and PTX3 is restricted to a specific subset of cells (34.5% in PDAC#1; 43.7% in PDAC#3) (**Figure 2F**, white arrows and **Figure 2G**, red arrows), compared to the neighbor cells which lack the expression of Ck-7 (**Figure 2F**, white arrowheads and **Figure 2G**, red arrowheads), thus suggesting in an attempt to speculate the identification of subpopulation of PDAC cells associated with high tumor metastatic potential.

### 3.3 Profiling of immune cells in PDAC

**Figure 3:**
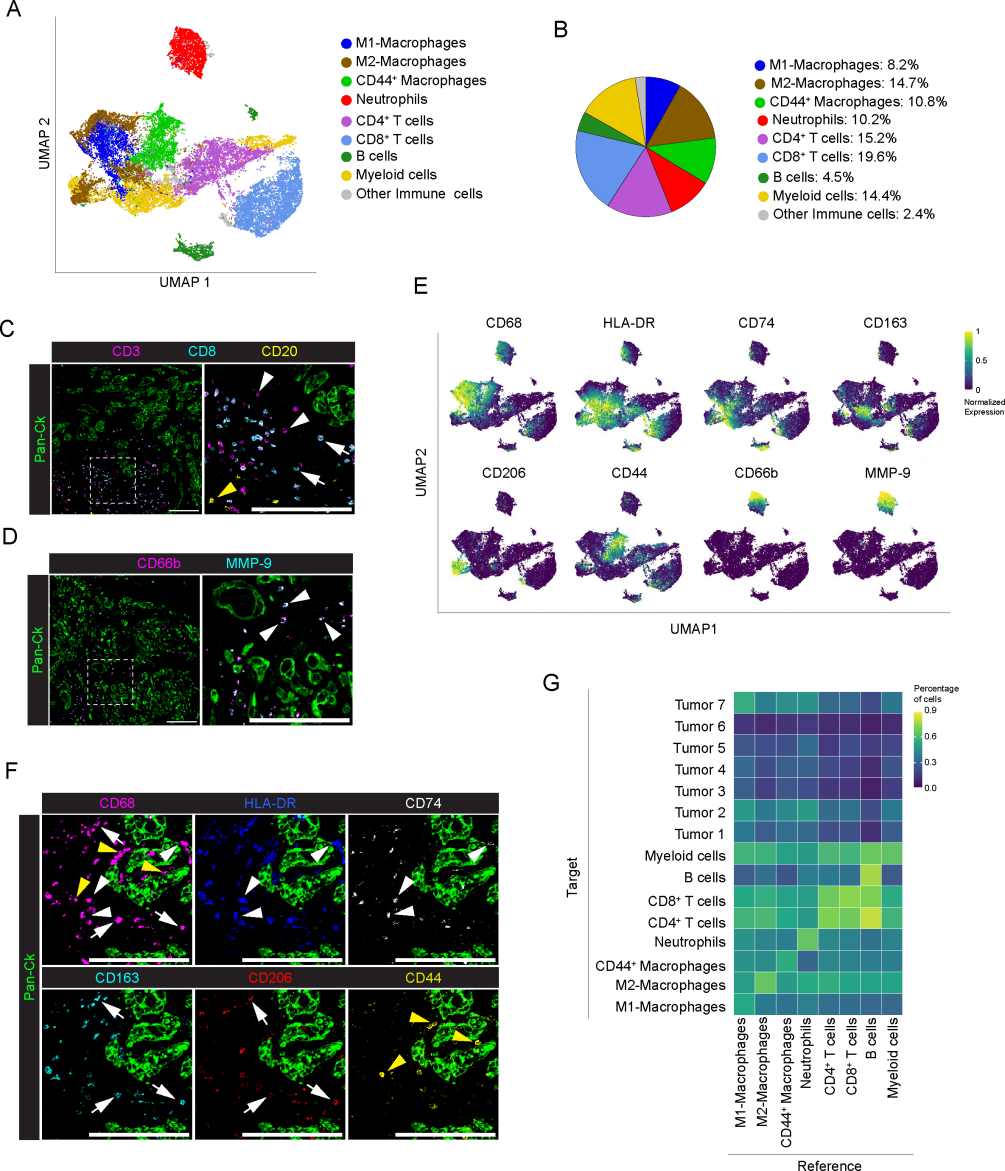
Profiling of immune cells in PDAC. **(A)** UMAP representation of immune cells in PDAC (n=8 PDAC), as in legend. **(B)** Frequency of cells belonging to the identified immune cell subpopulations, over the total number of immune cells, as in legend. **(C)** Representative images out of 34 acquired ROIs (n=8 PDAC) showing signal contribution of Pan-Ck (green) CD3 (magenta) CD8 (cyan) and CD20 (yellow). White arrowheads, white arrows and yellow arrows in the inset indicate CD4 T cells (identified as CD3^+^/CD8^-^ T cells), CD8^+^ T cells and B cells respectively. Bar: 200µm. **(D)** Representative images out of 34 acquired ROIs (n=8 PDAC) showing Pan-Ck (green), CD66b^+^ neutrophils (magenta) and MMP-9^+^ (cyan). Arrowheads in the inset indicate CD66b^+^ neutrophils expressing MMP-9. Bar: 200µm. **(E)** UMAP representation of the normalized expression of immune cell markers for the identification of macrophages subpopulation (M1 macrophages as CD68^+^/HLA-DR^high^/CD74^high^, M2 macrophages as CD68^+^/CD163^high^/CD206^high^ and CD44^+^ macrophages as CD68^+^/CD44^+^) and neutrophils (CD66b^+^/MMP-9^+^). **(F)** Representative images out of 34 acquired ROIs (n=8 PDAC) showing the extracted signal contribution of Pan-Ck (green), CD68 (magenta), HLA-DR (blue) CD74 (gray), CD163 (cyan), CD206 (red) and CD44 (yellow). White arrowheads indicate M1 macrophages as CD68^+^/HLA-DR^high^/CD74^high^, white arrows indicate M2 macrophages as CD68^+^/CD163^high^/CD206^high^, yellow arrows indicate CD44^+^ macrophages as CD68^+^/CD44^+^. Bar: 200µm. **(G)** Results of the neighboring cell analysis, as heatmap, showing the average percentage of each indicated cell subtype (Reference) that are in proximity (≤ 30μm distance) to each indicated cell subpopulations (Target).

In immune cell population (n=28661 cells), a functional specialization of macrophage was defined based on the expression of classical M1 (CD68^+^, HLA-DR^+^, CD74^+^) or M2 (CD68^+^, CD163^+^, CD206^+^) markers. The percentage of M2-like macrophages (14.7%, range 6.5-26.8% per PDAC) was higher compared to M1-like macrophages (8.2%, range 1.7-24.4% per PDAC), thus indicating a propensity for M2 deviation in the tumor microenvironment of PDAC, and hence sustained tumorigenesis, immune evasion, and metastasis formation (46, 47) (**Figures 3A, B**). A distinguished cluster of macrophages showed exclusive enrichment in CD44 (10.8%, range 1.5-35.9% per PDAC), and lower expression of HLA-DR and CD74, or of CD163 and CD206 (**Figures 1H**, **3E, F**, yellow arrowheads). In PDAC, the same macrophage subtype was recently described to belong to the vascular niche and to be distinguished by a pro-angiogenic gene signature (24). Noteworthy, distribution of macrophages around Pan-Ck tumor cells is shown in **Figure 3F**, with M2-like macrophages expressing higher levels of CD163 and CD206 (**Figure 3F**, white arrows) and M1-like macrophages expressing higher levels of HLA-DR and CD74 (**Figure 3F**, white arrowheads). In addition, as previously shown in **Figure 1H**, S100A4 expression was higher in M2-like macrophages compared to M1-like macrophages, suggesting their pro-tumorigenic activity (48). The remaining subtypes of immune cells identified were found to be CD66b^+^ neutrophils; CD3^+^, CD4^+^ (CD8^-^) T cells; CD3^+^, CD8^+^ T cells; CD20^+^ B cells; and eventually CD68^-^, CD206^+^ myeloid dendritic cells (**Figure 3A**; **Supplementary Figures 4A, B**). T cells represent the 34.8% of the tumor infiltrating cells (range 19.7-51.3%; n=8 PDAC), with CD8^+^ and CD4^+^ T cells counting for the 19.6% (range 8.4-32.2%; n=8) and 15.19% (range 5.1-26.2%; n=8) of T lymphocytes, respectively (**Figures 3B, C**: CD3^+^, CD8^-^, white arrowheads, CD3^+^, CD8^+^, white arrows). On the contrary, B cells poorly infiltrate tumor tissue (4.46%; range 0.1-13.3%; n=8) (**Figures 3B, C**; yellow arrowheads). Neutrophil cluster (10.2%; range 0.2-29.3%; n=8) (**Figure 3B**) infiltrating tumor tissues were identified based on the expression of CD66b and high levels MMP-9 stored into their tertiary granules (**Figures 3D, E**). As observed in tumor cell profiling, immune cell composition of the TME is heterogeneous among the analyzed patients: neutrophil abundance was higher in PDAC #1, (29.4%) #2 (20.5%), #3 (9.8%) and #6 (15.9%); PDAC #2 showed a highest frequency of both M1-like (24.4%) and M2-like (26.8%) macrophages; CD44^+^ were abundant in PDAC #4 (35.9%) and #8 (19.5%) (**Supplementary Figure 4C**).

Analysis of the frequency of cells (Reference) in close proximity (<30μm) with other cell types (Target) showed a preferential neighboring of B cells to both CD4^+^ (72 ± 28% of B cells) and CD8^+^ T cells (81 ± 21% of B cells) whereas no interaction was observed between immune cells and PDAC, with the exception of a weak association with CD44^+^ macrophages (59 ± 24% of cells *vs* all the Tumor subtypes), M1-like macrophages (67 ± 20% of cells *vs* all the Tumor subtypes) and neutrophils (65 ± 25% of cells *vs* all tumor subtypes) (**Figure 3G**; **Supplementary Figure 5**).

### 3.4 Profiling of CAFs in PDAC

**Figure 4:**
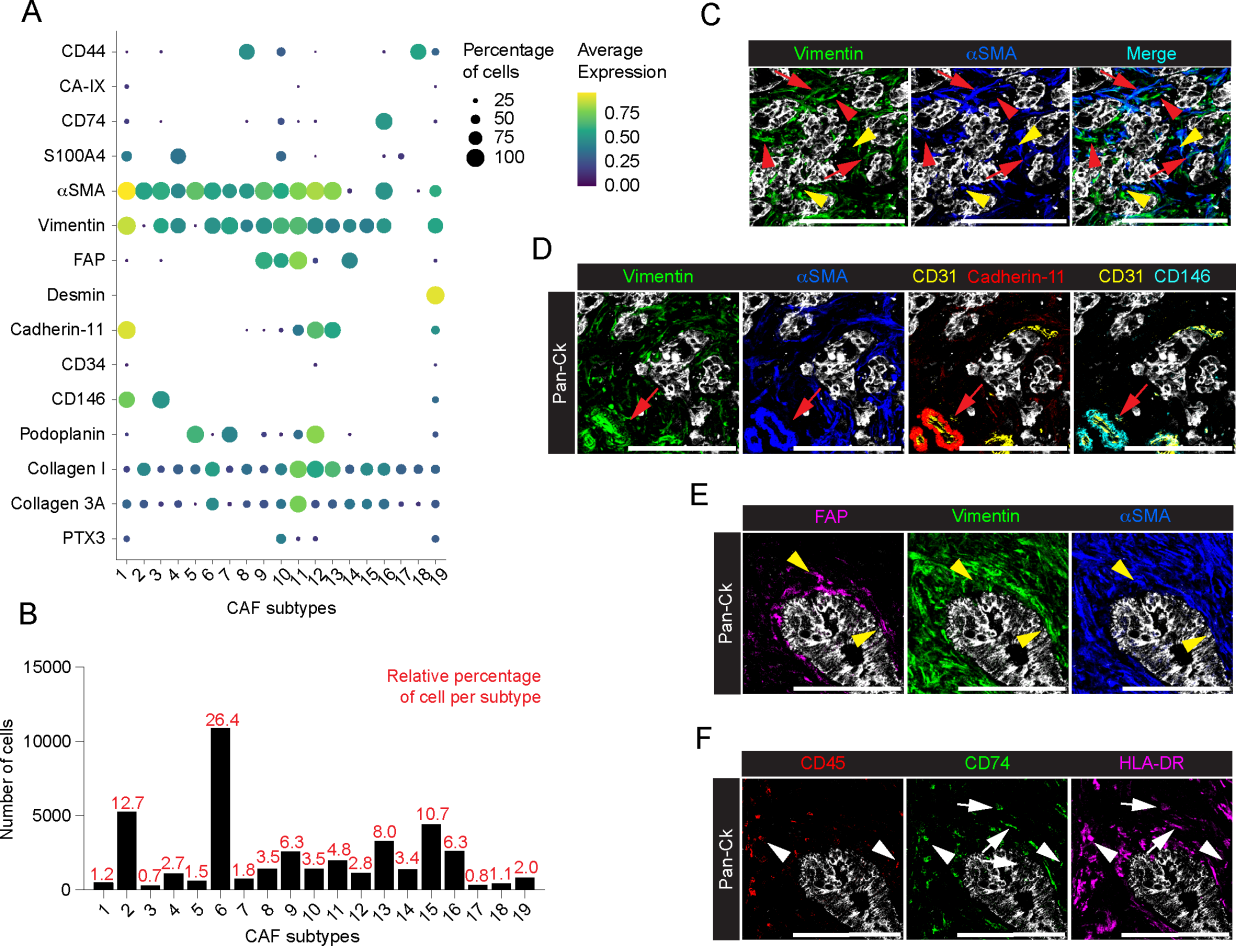
Profiling of CAFs in PDAC. **(A)** Dot plot showing each CAF marker average normalized expression and the relative percentage of positive cells, among the annotated CAF subtypes. **(B)** Barplot showing the relative percentage of cells among the identified subtypes on total CAFs. **(C)** Representative images out of 34 acquired ROIs (n=8 PDAC) of Pan-Ck (gray), vimentin (green) and αSMA (blue). Red arrows indicate cells co-expressing vimentin and αSMA. Red arrowheads and yellow arrowheads indicate cell expressing vimentin or αSMA only. Bar, 200µm. **(D)** Representative images out of 34 acquired ROIs (n=8 PDAC) showing the extracted signal contribution of Pan-Ck (gray), vimentin (green), αSMA (blue), CD31 (yellow), cadherin-11 (red) and CD146 (cyan). Red arrow indicates CD31^+^ blood vessel (yellow) covered by vimentin^+^/αSMA^+^/cadherin-11^+^/CD146^+^ pericytes. Bar, 200µm. **(E)** Representative images out of 34 acquired ROIs (n=8 PDAC) showing the extracted signal contribution of Pan-Ck (gray), FAP (magenta), vimentin (green) and αSMA (blue). Yellow arrowheads indicate CAFs co-expressing FAP, vimentin and αSMA surrounding Pan-Ck^+^ tumor cells. Bar, 200µm. **(F)** Representative images out of 34 acquired ROIs (n=8 PDAC) showing the extracted signal contribution of Pan-Ck (gray), CD45 (red), CD74 (green) and HLA-DR (magenta). White arrowheads indicate immune cells (CD45^+^) expressing CD74 and HLA-DR, while white arrows indicate CAFs (CD45^-^) expressing CD74 and HLA-DR. Bar: 200µm.

CAFs are tumor-promoting cells abundant in the ECM with a multifaceted phenotype (49, 50) and promising targets for new anticancer interventions (11, 51). In the present study, major efforts were therefore directed towards the identification of profiling markers of the different functional activities of CAFs in PDAC. Single-cell segmentation and PhenoGraph analysis identified n=41339 total CAFs (33.8% on total cells, n=8 PDAC; range 21-43.5% per PDAC tissue) annotated into 19 different subtypes, based on the differential expression of CD44, CA-IX, CD74, S100A4, αSMA, vimentin, FAP, desmin, cadherin-11, CD34, CD146, podoplanin, collagen I, collagen 3A and PTX3 (**Figure 4A**; **Supplementary Figure 5A**). As with immune cells, a phenotypic heterogeneity in the CAF population was found among the analyzed patients, although no specific CAF subtype was restricted to individual patients (**Supplementary Figure 5B**). According to the literature (49), most of the CAFs identified belonged to clusters 2 (12.7%, n=8; range 4.6-44.4%), 6 (26.4%, n=8; range 7-43.6%) and 15 (10.7%, n=8; range 2.4-30.6%) which express almost exclusively αSMA and vimentin (**Figures 4A, B**) and correspond to myofibroblast-like CAFs (myCAFs). In a further dissection of myCAFs, while CAFs 6 show a concomitant expression of αSMA and vimentin (**Figures 4A, C**, red arrows), CAFs 2 and CAFs 15 were expressing αSMA or vimentin alone, respectively (**Figures 4A, C**, red arrowhead: αSMA^+^ CAFs 2; yellow arrowhead: vimentin^+^ CAFs 15). Distinctly, the αSMA^+^ vimentin^+^ clusters CAFs 1 (1.2%, n=8; range 0.2-2.5%) and CAFs 3 (0.7%, n=8; range 0.2-2.9%) were patently identified as pericytes, exhibiting the expression of markers of mesenchymal origin, included cadherin-11 and CD146, and are associated CD31^+^ ECs (**Figure 4D**). In addition to the expression of αSMA and vimentin, CAFs 9 (6.3%, n=8; range 0.04-20.4%), 10 (3.5%, n=8; range 0.4-9.2%), 11 (4.8%, n=8; range 0.2-18%) and 14 (3.4%, n=8; range 0.3-12.9%) showed higher expression of FAP, also in combination with S100A4 and PTX3 (CAFs 10) or cadherin-11 (CAFs 11), thus identifying phenotypically different subtypes and emphasizing their functional evolution and plasticity in PDAC (e.g. myCAF *vs*. inflammartory CAFs (iCAFs)) (52, 53) (**Figure 4E**). Interestingly, FAP^+^ αSMA^+^ subtypes were localized closer to tumor cells compared to the previously identified myCAFs (**Figure 4E**) (average minimal distance from the tumor cells: CAFs 9, 29.7 ± 25.5µm, n=2591 cells; CAFs 10, 36.6 ± 60.2µm, n=1437 cells; CAFs 11, 32.5 ± 36.9µm, n=1980 cells; *vs*. CAFs 2, 68.0 ± 63.3µm, n=5254 cells; CAFs 6, 95.7 ± 108.0µm, n=10905 cells; CAFs 15, 61.3 ± 79.7µm; n=4430 cells; p-value < 10^-15^ KS test for all conditions), as well as to the FAP^+^ αSMA^-^ CAFs 14 subpopulation (56.4 ± 54.7µm, n= 1389; p-value < 10^-15^ KS test). Other subtypes identified included CAFs 19 distinguished by higher expression of desmin (2.0%, n=8; range 0.3-4.7%) (**Figures 4A, B**); CAFs 16 (6.3%, n=8; range 3.2-9.2%), the only cluster that showed a distinctive expression of CD74 and HLA-DR but lacking of CD45 expression (**Figure 4F**, white arrows), thus suggesting the overt identification of CAFs having immunological properties in PDAC, the so-called antigen-presenting CAFs (apCAFs) (54); the poorly represented CAFs 18 (1.1%, n=8; range 0.04-4.7%) and CAFs 8 (3.5%, n=8; range 0.3-6.7%) expressed high levels of CD44, alone (CAFs 18), or in combination with elevated αSMA and Vimentin (CAF 8) (**Figures 4A, B**); CAFs 5 (1.5%, n=8; range 0-7.7%), CAFs 7 (1.8%, n=8; range 0.2-7.2%) and CAFs 12 (2.8%, n=8; range 0-15.2%) showed expression of podoplanin, a well-defined CAF predictive marker of PDAC progression (55), in combination with αSMA (CAFs 5), αSMA and vimentin (CAFs 7), or with αSMA and vimentin and cadherin-11 (CAFs 12), (**Figures 4A, B**). In contrast to proteomic studies on CAFs in breast cancer (56), no consistent association between CD34 expression and CAFs was observed (**Figure 4A**). As expected, many of the clusters of CAFs that included CAFs 2, 6, 11, 12 and 13 (**Figure 4A**), were associated with collagen I and 3A, pointing to them as major players involved in a continuous interaction with the regions of the tumor tissue associated with deposition and remodeling of ECM (57).

**Figure 5:**
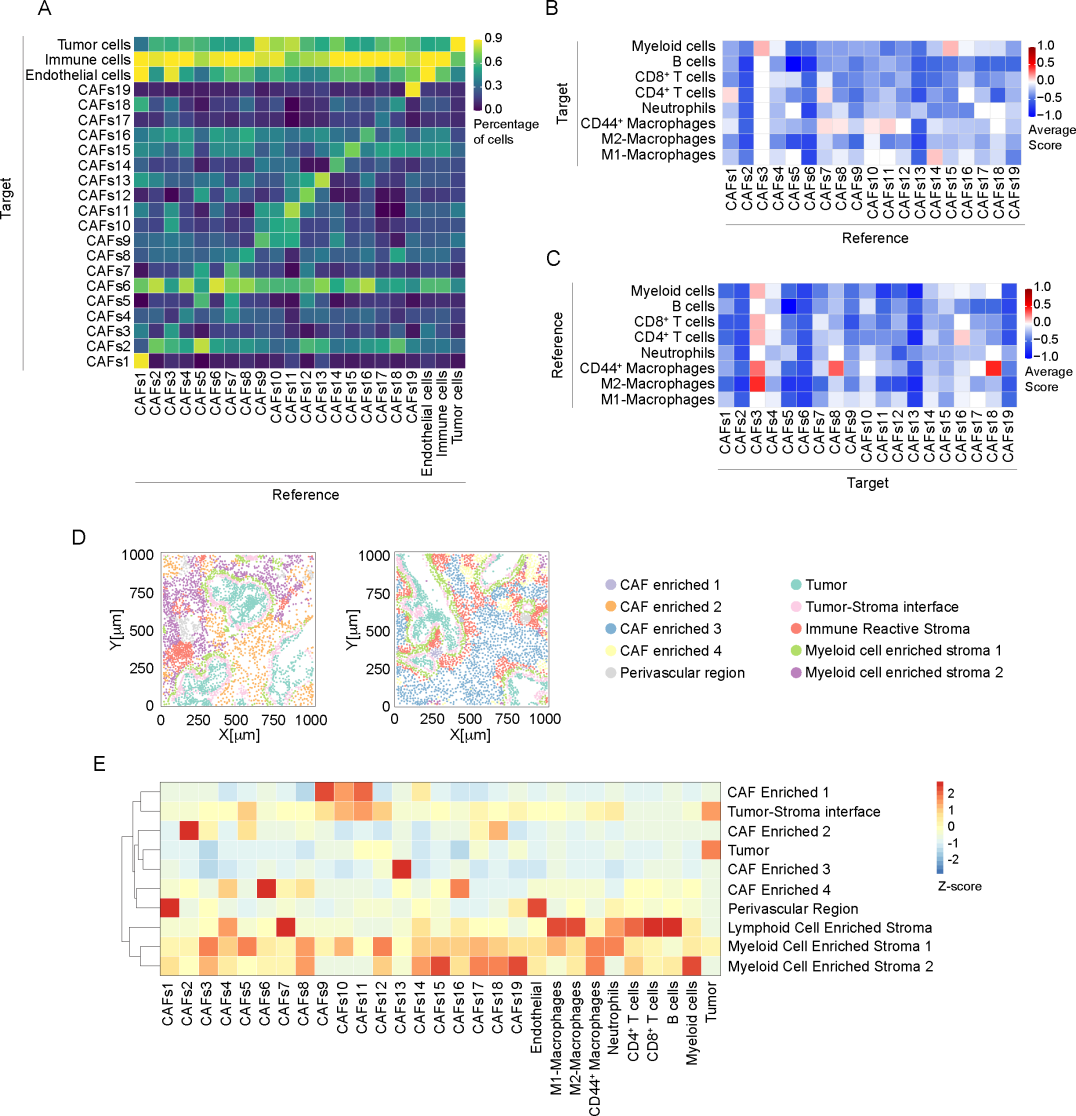
Neighborhood analysis of CAFs in PDAC. **(A)** Results of the single cell neighboring analysis, as heatmap, showing the average percentage of each indicated cell subtype (Reference) that are in proximity (≤ 30μm radius) to cells of each indicated subpopulation (Target). **(B)** Heatmap showing the average proximity score for each pair of cell phenotypic subpopulations of CAF (Reference) to immune cells (Target). Positive (red) or negative (blue) values indicate that a specific pair of phenotypes is neighboring significantly more often or significantly less often, respectively, than expected from a randomized placement, as described in Material and Methods. 30μm radius is considered for cell-to-cell proximity, as in **(A)**. **(C)** Heatmap, showing the average proximity score for each pair of cell phenotypic subpopulations, as in B, assuming immune cells as Reference populations and CAF subtypes as Target. **(D)** Representative spatial distribution of cellular neighborhoods, identified as regions with similar cellular composition, as described in Material and Methods, into two different ROIs (out of n=34). Each dot represents the center of mass of a single cell, dot color corresponds to annotated neighborhood, as in legend. Annotation was performed based on enrichment analysis reported in **Supplementary Table 3**. **(E)** Relative abundance of cellular populations across the annotated cellular neighborhoods as in **(D)** Values are normalized by column.

A spatial association between cell subpopulations was evaluated by neighboring analysis, highlighting pairwise association between specific cell types, and neighborhood enrichment, aimed to identify larger regions homogeneous in cell composition (**Figure 5**). In association with a high phenotype diversity of CAFs, neighboring cell analysis revealed the presence of high heterogeneity in their spatial relationship with other cells in PDAC (**Figure 5A**). Except for CAFs 6, which showed an association with CAFs 2, 4, 8, 13 and 16 (fraction of CAFs 6 neighboring CAFs 2, 51 ± 27%; CAFs 4, 30 ± 19%; CAFs 8, 34 ± 18%; CAFs 13, 36 ± 23%; CAFs 16, 50 ± 20%; n= 10905 cells), no specific relationship was found between them. As expected, CAFs 1 and 3, identified as αSMA^+^ vimentin^+^ CD146^+^ pericytes, (**Figures 4A, D**) were spatially associated with ECs (**Figure 5A**). In particular, CAFs 1 significantly associated with CD146^+^ and collagen IV^+^ CD31^+^ ECs (**Figure 1E**; **Supplementary Figure 5**, score 0.57 ± 0.51), thus suggesting a role in controlling blood vessel functionality.

In addition, CAF FAP^+^ subtypes (CAFs 9, 10 and 11) preferentially localized in proximity with Pan-CK+ cells (CAFs 9 86 ± 10%, n=2575 cells; CAFs 10 76 ± 19%, n=1405 cells; CAFs 11 80 ± 17%, n=1929 cells) (**Figure 5A**).

A consistent spatial association between CAFs and immune cells was observed (**Figure 5A**). Considering the subtypes of immune cells identified (**Figures 5B, C**), CAFs 1 and CAFs 3, identified as pericytes, showed an interaction, respectively, with CD4^+^ T cells (**Figure 5B**, score 0.07 ± 0.47) and myeloid cells (**Figure 5B**, score 0.14 ± 0.38). Moreover, CAFs 3 were associated with CD44^+^ macrophages (**Figure 5C**, score 0.29 ± 0.49) and M2-like macrophages (**Figure 5C**, score 0.43 ± 0.53). Of note, CAFs 1 and 3 mainly differ by the expression of cadherin-11 (**Figure 1E**), whose expression has been associated to anti-tumor immune response in a genetic mouse model of PDAC (58). Similarly, podoplanin^+^ CAFs 7 were found in association with CD4^+^ T cells (**Figure 5B**, score 0.06 ± 0.44) and CD44^+^ macrophages (**Figure 5B**, 0.06 ± 0.25). FAP^+^ CAFs 10 and 11 interacted preferentially with CD44^+^ macrophages (**Figure 5B**, CAFs 10, score 0.04 ± 0.36; CAFs 11, score 0.1 ± 0.3) while FAP^+^ CAFs 14 showed relationship with M1-like macrophages (**Figure 5B**, score 0.11 ± 0.47). These CAF subtypes were observed in the peritumoral niche around Pan-Ck^+^ cells, in correspondence with an enrichment of macrophages (**Figures 3H**, **4G, E**). As evidence for an identification of a CAF cluster with immunoregulatory properties (apCAFs) in PDAC, CD4^+^ T cells were observed significantly close to CAFs 16 (**Figure 5C**, score 0.09 ± 0.445).

We then investigated the presence of patterns of localized enrichment resulting in the identification of 10 classes of regions similar in cellular composition (neighborhoods, **Figures 5D, E**). Thus, each ROI can be divided into sub-regions highlighting spatial adjacent cells belonging to different spatial context (59) (**Figure 5D**). The analysis shows that regions identified as tumor-stroma interface are particularly enriched of FAP^+^ CAFs 10 and CAFs 11 (*padj<0.001*, **Figure 5E**; **Supplementary Table 3**), in agreement with the evidence resulting from the neighboring analysis (**Figures 4E**, **5A**). In addition, CAFs 1, expressing pericyte markers, were enriched in perivascular region (*padj<0.001*). We also identified three neighborhoods of immune cells, enriched either in B cells and CD8^+^ T cells (Lymphoid Cell Enriched Stroma), or neutrophils and CD44^+^ and M1-like macrophages (Myeloid Enriched Stroma 1) or myeloid cells and CD44^+^ macrophages (Myeloid Enriched Stroma 2), with distinct spatial distribution in the ROIs (**Figures 5D, E)**. Several CAF subtypes were associated with the immune enriched neighborhoods. Among them, apCAFs (CAFs 16) were associated with Lymphoid Cell Enriched Stroma and Myeloid Cell Enriched Stroma 2 (*padj<0.001*, **Figure 5D**; **Supplementary Table 3**).

**Figure 6:**
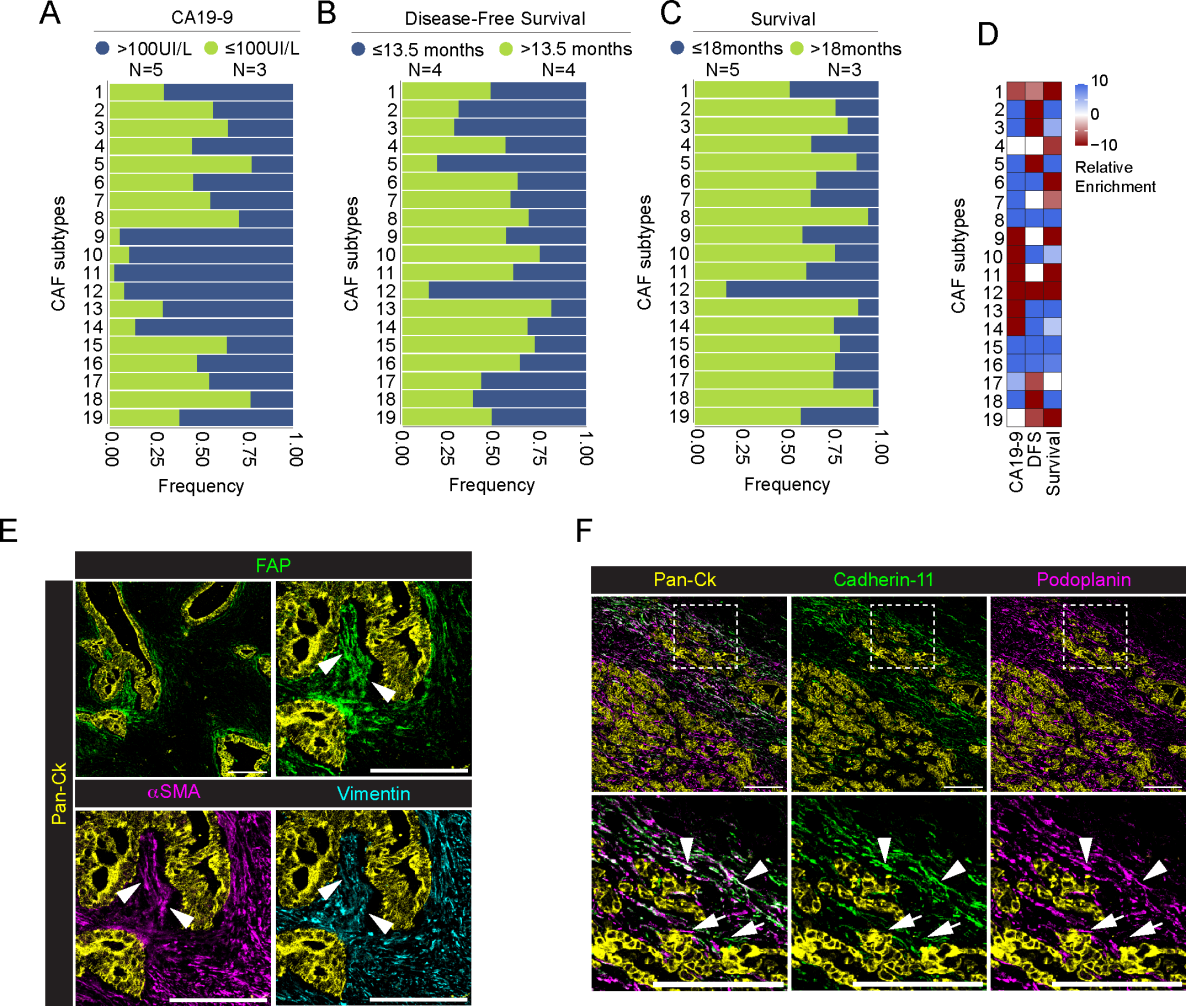
Association between CAFs and patients’ levels of CA19-9, disease-free survival and survival status. **(A-C)** Barplot showing frequency of each CAF subpopulation in association with patients’ pre-operative levels of CA19-9 [**(A)**, blue >100IU/l, green ≤100IU/l], disease-free survival [**(B)**, blue ≤13.5 months, green >13.5 months] and overall survival [**(C)**, blue ≤18 months, green >18 months) (PDAC n=8 total)]. Data are reported as frequency normalized over the total number of cells in each CAF group and annotated according to patient status. Number of patient in each group is reported in legend. **(D)** Relative enrichment score of each CAF subtype in the good (red) or bad (lightblue) prognosis group for three different clinical parameters as above. Higher absolute values correspond to more significant enrichment, values closer to zero are less significant. P-value adjusted > 0.001 are set to enrichment 0. **(E)** Representative images out of 34 acquired ROIs (n=8 PDAC) showing the extracted signal contribution of Pan-Ck (yellow), FAP (green), αSMA (magenta) and vimentin (cyan). White arrowheads indicate CAFs co-expressing FAP, vimentin and αSMA surrounding Pan-Ck^+^ tumor cells. Bar, 200µm. **(F)** Representative images out of 34 acquired ROIs (n=8 PDAC) showing the extracted signal contribution of Pan-Ck (yellow), cadherin-11 (green) and podoplanin (magenta) (Upper panel). White arrows and arrowheads in close up images (lower panel) show podoplanin^+^ CAFs and cadherin-11^+^ podoplanin^+^ CAFs, respectively. Bar, 200µm.

To evaluate an association between CAF subtypes and PDAC progression, we evaluate their distribution in patients with circulating levels of carbohydrate antigen 19-9 (CA19-9) (60), disease-free survival (DFS) and survival status (**Supplementary Table 1**). We divided patients into high CA19-9 (n=5) and low CA19-9 (n=3), setting a threshold level of 100IU/L, as previously reported (61). We found that FAP^+^ CAFs, namely CAFs 9, CAFs 10, CAFs 11 and CAFs 14, as well as podoplanin^+^ cadherin-11^+^ CAFs12 were enriched in patients with higher level of CA19-9 (*padj<0.001*) (**Figures 6A, D–F**; **Supplementary Figure 6A**). Interestingly, CAFs 12, as well as podoplanin^+^ CAFs 5 are also associated with a worst DFS (n= 4 patients, DFS<13.5 months, *padj <0.001*) (**Figures 6B, D**; **Supplementary Figure 6B**). Similarly, CAFs 12 were associated to a shorter survival (n=3 patients, survival<18 months, *padj<0.001*)(**Figures 6C, D**; **Supplementary Figure 6C**). Higher frequency of CAFs 12 (81%) was found in the metastatic patient #1. **Figure 6F** show the concomitant infiltration of podoplanin^+^ cadherin-11^-^ CAFs 5 (white arrow) and podoplanin^+^ cadherin-11^+^ CAFs 12 (white arrowheads), close to tumor cells. Finally, CAFs 1, expressing the conventional pericyte markers, are associated with higher CA19-9 levels and worst patients prognosis (*padj<0.001*)(**Figures 6A-D**; **Supplementary Figure 6**). On the other hand, CD44^+^ CAFs 8, vimentin^+^ CAFs 15 and apCAFs (CAFs 16) are associated with lower CA19-9 levels, as well as longer DFS and survival (*padj<0.001*) (**Figures 6A-D**; **Supplementary Figure 6**).

Overall, single-cell resolution IMC analyses shed light on the phenotypic and spatial complexity of associated PDAC infiltrating CAFs, reflecting their possible functional differences that contribute to disease progression.

## 4 Discussion

Over the last years, several transcriptomic studies described the heterogeneity of CAFs in PDAC, underlying their pivotal role in disease progression and resistance to therapy. Analyses of gene expression profile and spatial location led to the identification of three main CAF subtypes, named myCAFs, iCAFs and apCAFs (52, 62, 63). Although transcriptomic approaches have the potential to identify thousands of genes and new signatures in tissues, a correlation between mRNA and protein can be limited by several factors, including post-transcriptional machinery (64). For this reason, several studies combined single-cell RNA analysis and multiplexed imaging to provide a comprehensive analysis of diseased tissue. Recent studies report the relevance of using IMC complementary to transcriptomics for the purpose of defining the phenotype of cell subpopulations previously revealed by transcriptional approaches (54, 56, 65). On the other hand, while transcriptomic analysis has provided important information on the molecular pathways involved in the activation and differentiation of CAFs in PDAC, only a few studies have investigated their phenotype profiling by protein detection.

In the present work, we applied IMC to investigate cellular composition of 8 PDAC patients. By using a 31-antibody panel, IMC allowed us to describe the tissue architecture, identifying different subtypes of cancer cells, immune cells, ECs and CAFs. In addition, neighborhood analysis implemented the phenotypic data, providing information about the spatial localization of distinct cellular subtypes and their relationship within tumor tissue.

PDACs are characterized by a marked degree of both inter-tumor and intra-tumor heterogeneity in the histomorphology of tumor cells (66). These morphological differences coexist in the same tissues and are associated with distinct gene profiles and generally correlate with disease outcome (67, 68). In addition, morphological heterogeneity can be randomly observed even between immediately adjacent tumor cells, with a gradual transition from one morphological type to another (66). Recently, the concept of morpho-biotypes has been introduced to describe the diverse morphological and spatial organization of PDAC cells with different gene expression profiles, leading to the classification of PDAC into “glandular”, “transitional” and “undifferentiated” (69). In our IMC analysis, we identified 7 different tumor cell subtypes. Beside the common expression of Pan-Ck, tumor cells were characterized by the expression of markers associated with tumor progression, epithelial-to-mesenchymal transition, and resistance to therapy, such as CD44, S100A4 and CA-IX (41, 70, 71). As reported (72) we also observed a marked degree of inter-tumor variability, with some subtypes of tumor cells whose expression was limited to specific patients in our cohort. We identified a subset of tumor cells expressing Pan-Ck, Ck-7 and PTX3, associated with the only two patients in our cohort having distant metastasis at the time of the diagnosis and surgery. Interestingly, a cancer-derived PTX3 production has been associated with tumor progression in several type of cancer, including melanoma, cervical cancer, hepatocellular carcinoma and glioma (44, 45, 73, 74), possible consequence of its role in remodeling ECM (75–77) that occurs in acidic microenvironments (76), an hallmark in cancer (78).

PDAC is generally considered an “immunologically cold” tumor, showing intrinsic properties that lead to the evasion of an effective immune response (79, 80). Almost 90% of PDACs show mutation in KRAS, which is associated to the secretion of granulocyte-macrophages colony-stimulating factor (GM-CSF) and the consequent recruitment of immunosuppressive myeloid cells, as well as the upregulation of programmed death ligand-1 (PD-L1) (81, 82). In addition, the recruitment of regulatory immune cells and the secretion of chemokine and cytokine, such as CXCL12, IL-10 and TGFβ, contribute to the generation of an immunosuppressive microenvironment (83). However, we identified that PDAC is predominantly accompanied by infiltration of M2-like macrophages, consistent with their association with tumor progression, recurrence and metastatic spread in PDAC patients (46). In addition, we identified a distinct population of CD44^+^ macrophages. Recently, CD44^+^ macrophages have been described in PDAC tissue as a subtype of HLA-DR^Low^ macrophages enriched in the vascular niche and possibly promoting neovascularization (24). Our analysis confirmed the presence of this phenotype, with lower HLA-DR expression compared to M1-like macrophages: differently, we found that, compared to the other immune cells, CD44^+^ macrophages, together with M1-like macrophages and neutrophils, are the only immune cells showing a weak spatial interaction with tumor cells. Moreover, our neighborhood analysis (**Figures 3G**, **5D**) showed that immune cells are generally excluded from tumor cell-enriched areas, confirming the immune suppressive microenvironment of PDAC tissue.

As widely described (12, 49, 84), CAFs play a pivotal role in the PDAC development and therapeutic response, by regulating the composition and the structure of the ECM, contributing to the generation of an immune-suppressive environment and influencing PDAC cell proliferation, invasion and drug resistance (11, 84). CAFs have been classified on the basis of their phenotype and function into myCAFs, having high expression of αSMA and located in proximity to tumor cells, iCAFs, showing IL-6^high^ inflammatory feature and located away from neoplastic cells, and apCAFs, expressing MHC-II and CD74, whose function is still matter of study (49, 54). Beside this classification, a variety of markers have been used to define CAFs, but most of them are shared among CAF and non-CAF cell type, thus requiring a further identification of subtype specific markers (85). In our study, we combined the expression of 11 established CAF markers (56, 65), resulting in the definition of 19 different CAF subtypes having distinct phenotype, tissue localization and relationship with other cells. We found that the vast majority of CAFs expressed αSMA and vimentin, thus suggesting the identification of the myCAF phenotype. αSMA^+^ Vimentin^+^ myCAFs can be further distinguished into distinct subtypes, based on the differential expression of other CAF markers, such as CD146, S100A4, podoplanin, FAP, CD44 and cadherin-11. αSMA and vimentin can be concomitantly (CAFs 6) or alternatively expressed (CAFs 2 and CAFs 15). Recently, it has been demonstrated that higher vimentin expression in CAFs is associated with a significantly shorter patient survival. In addition, CAFs expressing vimentin alone, without the expression of αSMA, represent an independent predictor of poor prognosis (86). In addition to myCAFs, we identified a subtype of CAF expressing CD74 and HLA-DR (CAFs 16), likely corresponding to apCAFs (54). Unlike professional antigen-presenting cells, apCAFs did not express costimulatory molecules, such as CD40, CD80 and CD86: it has been hypothesized that MHC-II expressed by apCAFs might act as a decoy receptor for CD4^+^ T cells, inhibiting their clonal expansion, inducing anergy and promoting differentiation into T-regulatory cells, thus contributing to the generation of an immunosuppressive microenvironment (62, 87). In line with this observation, our neighborhood analysis revealed a spatial relationship between CD4^+^ T cells and CAFs 16 (apCAFs), suggesting a possible immunomodulatory activity.

Generally, CAFs localized in proximity to different immune cell-enriched regions associated with M2-like macrophages, CD44^+^ macrophages, T cells and myeloid cells. Recently, in mouse models of PDAC, is has been demonstrated that *Cdh11* deficiency alters the molecular profile of fibroblasts, reduces the expression of immunosuppressive cytokines and increases the anti-tumor immunity (58, 88).

Podoplanin has emerged as a robust marker for CAFs in PDAC, showing a correlation with worst patients’ prognosis (54, 89). More recently, it has been described a role of podoplanin-positive CAFs in the regulation of immune cell infiltration in PDAC and other tumors (90, 91). In our study, we identified 3 different CAF subtypes expressing podoplanin: among them, CAFs 7 also showed a significant spatial relationship with CD4^+^ T cells and CD44^+^ macrophages, suggesting a possible immune modulatory activity of this CAF population.

FAP is considered another well-defined CAF marker in PDAC. FAP^+^ CAFs are the main repository for CXCL12 in PDAC tumor microenvironment: CXCL12 promotes T cell spatial exclusion, and pharmacological inhibition of CXCL12-CXCR4 interaction results in T cell accumulation in tumor tissue and fostering of immune checkpoint blockade (92). In addition, FAP^+^ CAFs contribute to ECM desmoplasia, leading to the formation of a dense ECM which limit T cell proximity to PDAC tumor cells (93–96). In our analysis, we found 4 distinct CAF subtypes expressing FAP (CAFs 9, 10, 11 and 14): among this, CAFs 10 and 11 were the only CAF subtypes significantly enriched in tumor-stroma interface region and strictly associated to tumor cells. Moreover, we observed a significant spatial relationship between CAFs 10 and CD44^+^ macrophages, possibly regulating their recruitment and activity. Given the described role of FAP^+^ CAFs in regulating desmoplastic reaction and CD44^+^ macrophages as highly phagocytic cells, in an attempt to speculate these cells can cooperate to a ECM remodeling niche that promotes tumor cell invasion and PDAC progression (42, 94, 97).

CA19-9 is a validated marker to establish PDAC progression, with a good sensitivity (61, 98). Even if the number of patients included in this study is not sufficient to provide reliable correlation between CAF subtypes and patients prognosis, we report that CAFs expressing different combination of FAP, podoplanin and cadherin-11, were associated with higher level of CA19-9.

Recently, expression of αSMA by pericytes, induced by cancer cell-derived exosomes, has been associated with an alteration to the morphology and bio-mechanical properties of pericytes, which significantly correlate with vascular leakiness and hypoxia in PDAC (99), thus compromising the stability of tumor vasculature and hence affecting therapy efficacy. Interestingly, CAFs 1, which expressed higher levels of αSMA compared to the other identified pericyte subtype (CAFs 3), were enriched in perivascular regions, and resulted associated with a worst patients conditions.

In addition to CAFs 1, CAFs 12 cells, expressing podoplanin and cadherin-11, are associated with higher levels of CA19-9 as well as shorter DFS and overall survival. This result is in accordance with the recently observed association between the expression of podoplanin and cadherin-11 and the expansion of mesothelial cells that contribute to stromal deposition and desmoplastic reaction in early neoplastic lesions in mouse (87). In our cohort, majority of CAFs 12 were expressed by the metastatic patient#1, suggesting a possible correlation with PDAC metastatic capability. In line with this observation, podoplanin expression by CAFs has been associated with PDAC progression and invasion (55). On the other side, CAFs 15 and CAFs 16 are associated with lower CA19-9 levels and better patient prognosis. As previously reported, we identified CAFs 16 as apCAFs, based on the concomitant expression of CD74 and HLA-DR (54). Although apCAFs have been generally linked to the generation of an immunomodulatory microenvironment (62, 87), they may exert more complicated immune-regulating functions. For example, in breast cancer, MHC-II^+^ CAFs were associated with T regulatory cells and resistance to immunotherapy but also correlated with patient survival (100–102). In addition, in lung cancer, MHC-II^+^ CAFs enhanced CD4^+^ T cell cancer immunity (103).

In conclusion, our IMC analysis provided an overview of the complexity of the PDAC tumor microenvironments, showing how cancer and stromal cells with different phenotypic characteristics co-exist within the same tissue. In particular, the classification of 19 distinct CAF subtypes, characterized by different combination of fibroblast markers and by a peculiar spatial localization and relationship with surrounding cells, underlies the high plasticity of CAFs in PDAC and their complex role in PDAC progression, leading to the potential identification of new targets for the diagnosis and the treatment of PDAC patients.

## Data Availability

The raw data supporting the conclusions of this article will be made available by the authors, without undue reservation.
