# Supplementary material for: Depicting the cellular complexity of pancreatic adenocarcinoma by Imaging Mass Cytometry: focus on cancer-associated fibroblasts
Source: Front Immunol. 2024 Nov 7;15:1472433. doi: 10.3389/fimmu.2024.1472433 (PMC11578750; doi:10.3389/fimmu.2024.1472433)
Supplement: Supplementary file 1 [file DataSheet1.pdf]

Supplementary Material

Patient #	Sex	Age	G	T	LN Metastasis	Margin Resection	Distant MTS	LV invasion	Pn	CA19-9 (IU/L)	DFS	Overall Survival
#1	F	82	G2	pT3	N1	R1	M1	Lv1	Pn1	165.5	3	3
#2	F	84	G3	pT2	N2	R1	-	Lv1	Pn1	640.5	2	16
#3	F	75	G2	pT2	N1	R1	M1	Lv1	Pn1	166.7	16	20
#4	F	81	G2	pT2	N2	R1	-	Lv1	Pn1	45.3	11	21
#5	M	71	G2	pT3	N2	R1	-	Lv1	Pn1	56.3	22	22
#6	M	72	G3	pT2	N2	R1	-	Lv1	Pn1	148.1	8	12
#7	M	83	G2	pT3	N1	R1	-	Lv1	Pn1	404	20	20
#8	M	76	G2	pT2	N2	R1	-	Lv1	Pn1	15.6	19	19

Supplementary Table 1: Patients' pathological features. Sex: F: female; M: male; Grade: G1: well differentiated (>95% tumor glands), low grade; G2: moderately differentiated (50-95% tumor glands), low grade; G3: poorly differentiated (<50% tumor glands), high grade ; Tumor Dimension (T): pT2: 2-4 cm; pT3: > 4 cm; Lymph Node Metastasis: N1: 1-3 lymph node metastasis; N2: ≥ 4 lymph node metastasis; Margin Resection: R1: surgical margin are microscopically positive for residual tumor; Distant Metastasis: M1: distant metastasis in other organs, serosal or no locoregional lymph nodes. Lympho-vascular invasion: Lv1: presence of lympho-vascular tumoral invasion; Perineural invasion: Pn1: presence of perineural tumoral infiltration; CA19-9: levels of carbohydrate antigen (CA) 19-9, expressed as IU/L; DFS: Disease-free survival, expressed in months; Overall survival: patients' overall survival, expressed in months.

Target	Clone	Metal	Source
αSMA	1A4	141Pr	Standard Biotools
Peptide C	EPR22500-5	142Nd	Abcam
Vimentin	D21H3	143Nd	CST
CD74	D5N3I	144Nd	CST
CA-IX	EPR4151(2)	145Nd	Abcam
CD146	Polyclonal	146Nd	R&D Systems
CD163	EDHu-1	147Sm	Standard Biotools
Pan-Ck	C11	148Sm	CST
Fibrinogen	EPR1344	149Sm	DAKO
MMP-9	D6O3H	150Nd	CST
CD31	EPR3094	151Eu	Abcam
CD45	D9M8I	152Sm	CST
CD44	IM7	153Eu	eBioscience
S100A4	Polyclonal	154Sm	Merck Millipore
Collagen 3A	E8D7R	155Gd	CST
CD68	KP1	159Tb	Invitrogen
FAP	Polyclonal	160Gd	R&D Systems
CD20	H1	161Dy	Standard Biotools
CD66b	80H3	162Dy	Standard Biotools
PTX3	Polyclonal	163Dy	Home made (90)
Ck-7	RCK105	164Dy	Invitrogen
Desmin	Y66	165Ho	Abcam
Collagen IV	EPR20966	166Er	Abcam
CD8a	C8/144B	167Er	CST
CD206	Polyclonal	168Er	Abcam
Collagen I	Polyclonal	169Tm	Standard Biotools
CD3	Polyclonal	170Er	DAKO
Podoplanin	NC-08	171Yb	Biolegends
Cadherin-11	283416	172Yb	R&D Systems
CD34	EP373Y	175Lu	Abcam
HLA-DR	LN3	176Yb	eBioscience

Supplementary Table 2: Imaging Mass Cytometry antibody panel

Neighborhoods annotation	p-values	fdr	subpopulation
CAF Enriched 1 (n=4397)	0.E+00	0.E+00	CAFs9
	0.E+00	0.E+00	CAFs11
	6.E-168	1.E-166	CAFs10
	9.E-56	1.E-54	CAFs14
	9.E-07	4.E-06	Neutrophils
Tumor-stroma interface (n=21686)	0.E+00	0.E+00	Tumor
	3.E-38	3.E-37	CAFs11
	4.E-04	2.E-03	CAFs10
CAF Enriched 2 (n=4854)	0.E+00	0.E+00	CAFs2
	2.E-50	2.E-49	CAFs18
	8.E-45	8.E-44	CAFs5
	2.E-10	1.E-09	CAFs17
	5.E-09	3.E-08	CAFs3
	1.E-08	5.E-08	CAFs8
	2.E-07	8.E-07	CAFs16
	2.E-06	7.E-06	Myeloid cells
Tumor (n=17297)	0.E+00	0.E+00	Tumor
CAF enriched 3 (n=3144)	0.E+00	0.E+00	CAFs13
	1.E-19	9.E-19	CAFs16
	9.E-19	6.E-18	CD8+ Tcells
	1.E-12	8.E-12	CAFs12
	4.E-10	2.E-09	CAFs8
CAF enriched 4 (n=10872)	0.E+00	0.E+00	CAFs6
	9.E-112	1.E-110	CAFs16
	5.E-22	4.E-21	CAFs4
	2.E-13	9.E-13	CAFs8
	1.E-06	6.E-06	CAFs15
	1.E-04	5.E-04	CAFs7
	2.E-04	8.E-04	CAFs13
	2.E-03	7.E-03	CAFs2
Perivascular region (n=7128)	0.E+00	0.E+00	Endothelial cells
	9.E-150	2.E-148	CAFs1
	2.E-28	2.E-27	Neutrophils
	2.E-25	2.E-24	CAFs19
	2.E-18	1.E-17	CD4+ T cells
	2.E-17	1.E-16	M2-Macrophages
	3.E-08	1.E-07	B cells

frontiers

	1.E-05	4.E-05	CAFs14
	7.E-04	3.E-03	CAFs12
	9.E-04	3.E-03	CD8+ T cells
Immune reactive stroma (n=13720)	0.E+00	0.E+00	B cells
	0.E+00	0.E+00	CD8+ T cells
	0.E+00	0.E+00	M1-Macrophages
	0.E+00	0.E+00	M2-Macrophages
	4.E-287	7.E-286	CD4+ T cells
	4.E-99	6.E-98	Neutrophils
	6.E-86	7.E-85	CAFs7
	4.E-28	3.E-27	CAFs4
	2.E-13	1.E-12	Myeloid cells
	6.E-08	3.E-07	CAFs14
	6.E-06	3.E-05	CAFs16
Myeloid cell enriched stroma 1 (n=19900)	9.E-59	1.E-57	Neutrophils
	4.E-51	4.E-50	CD44+ Macrophages
	2.E-44	2.E-43	M1-Macrophages
	4.E-23	3.E-22	CAFs15
	5.E-18	3.E-17	CAFs12
	2.E-17	1.E-16	CAFs5
	1.E-08	7.E-08	CAFs8
	7.E-08	3.E-07	CAFs10
Myeloid cell enriched stroma 2 (n=13186)	0.E+00	0.E+00	CAFs15
	0.E+00	0.E+00	Myeloid cells
	3.E-139	5.E-138	CD44+ Macrophages
	7.E-94	1.E-92	CD4+ T cells
	5.E-58	6.E-57	CAFs19
	3.E-43	3.E-42	CAFs14
	3.E-32	3.E-31	CAFs8
	9.E-27	8.E-26	Endothelial cells
	7.E-22	5.E-21	CAFs18
	4.E-17	3.E-16	CAFs16
	1.E-16	7.E-16	CAFs12
	2.E-16	1.E-15	M2-Macrophages
	4.E-14	2.E-13	CAFs17
	7.E-12	4.E-11	B cells
	3.E-09	1.E-08	CAFs1
	2.E-07	7.E-07	CD8+ T cells
	4.E-05	2.E-04	CAFs4
	1.E-04	5.E-04	CAFs3

Supplementary Table 3: Subpopulation enriched for each cellular neighborhood. Single cells from PDAC patients (n=116184, over n=34 ROIs), were classified in ten clusters based on similarity of the set of neighboring cells (<30μm distance) using kmeans algorithm (see Materials and methods). Table shows p-values obtained from hypergeometric test and corresponding false discovery rate (fdr) for each subcellular population within the ten neighborhoods regions. Size of each group is also indicated.

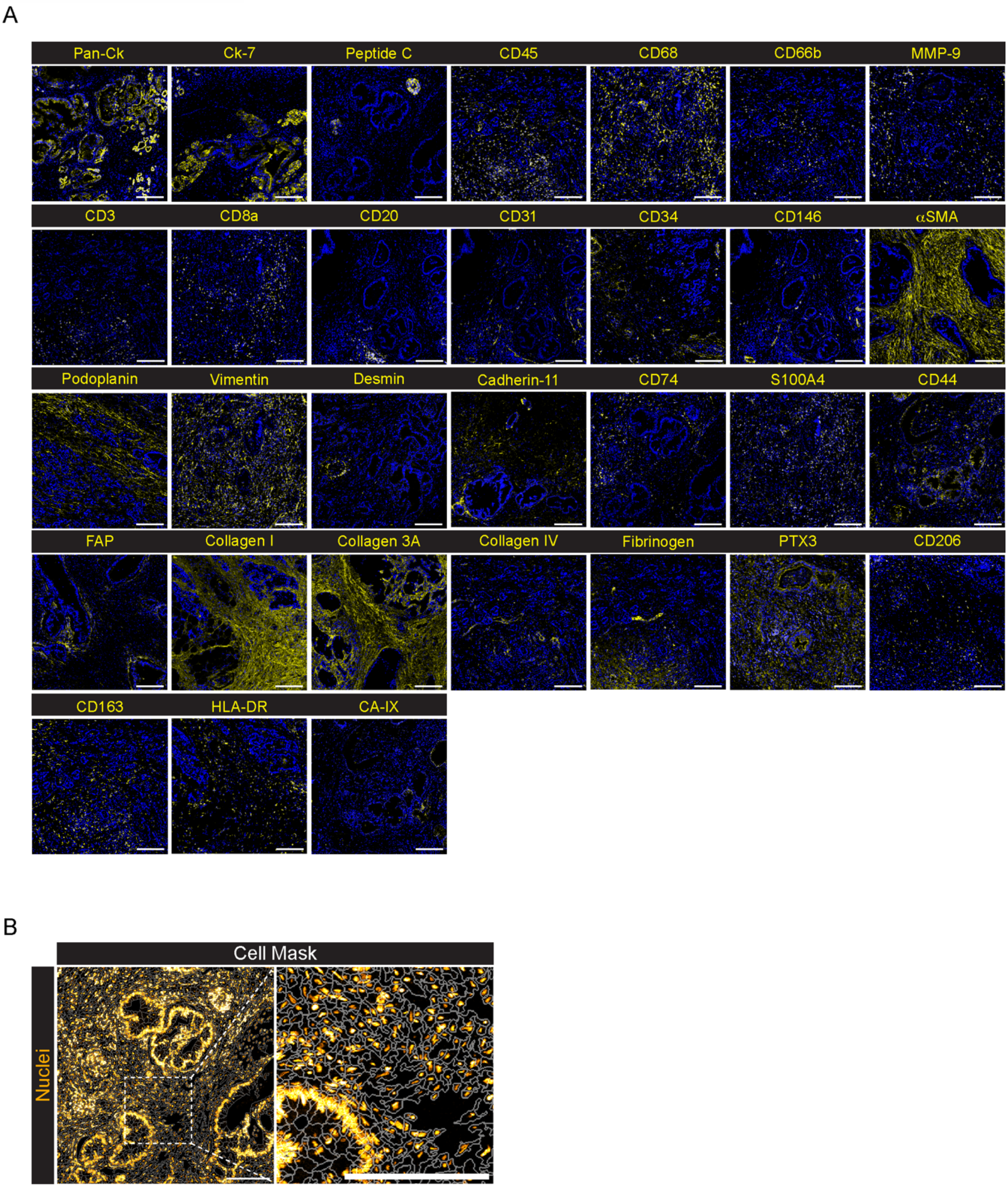

Supplementary Figure 1: Imaging Mass Cytometry image acquisition and segmentation. A) Representative images out of 34 acquired ROIs (n=8 PDAC) showing the extracted signal contribution of the 31 markers included in the Imaging Mass Cytometry panel. Marker and nuclei are shown in yellow and blue, respectively. Bar: 200µm. **B**) Representative image out of 34 acquired ROIs (n=8 PDAC) showing the result of the single-cell segmentation process. Yellow-hot: nuclei; gray: cell contours. Bar: 200µm.

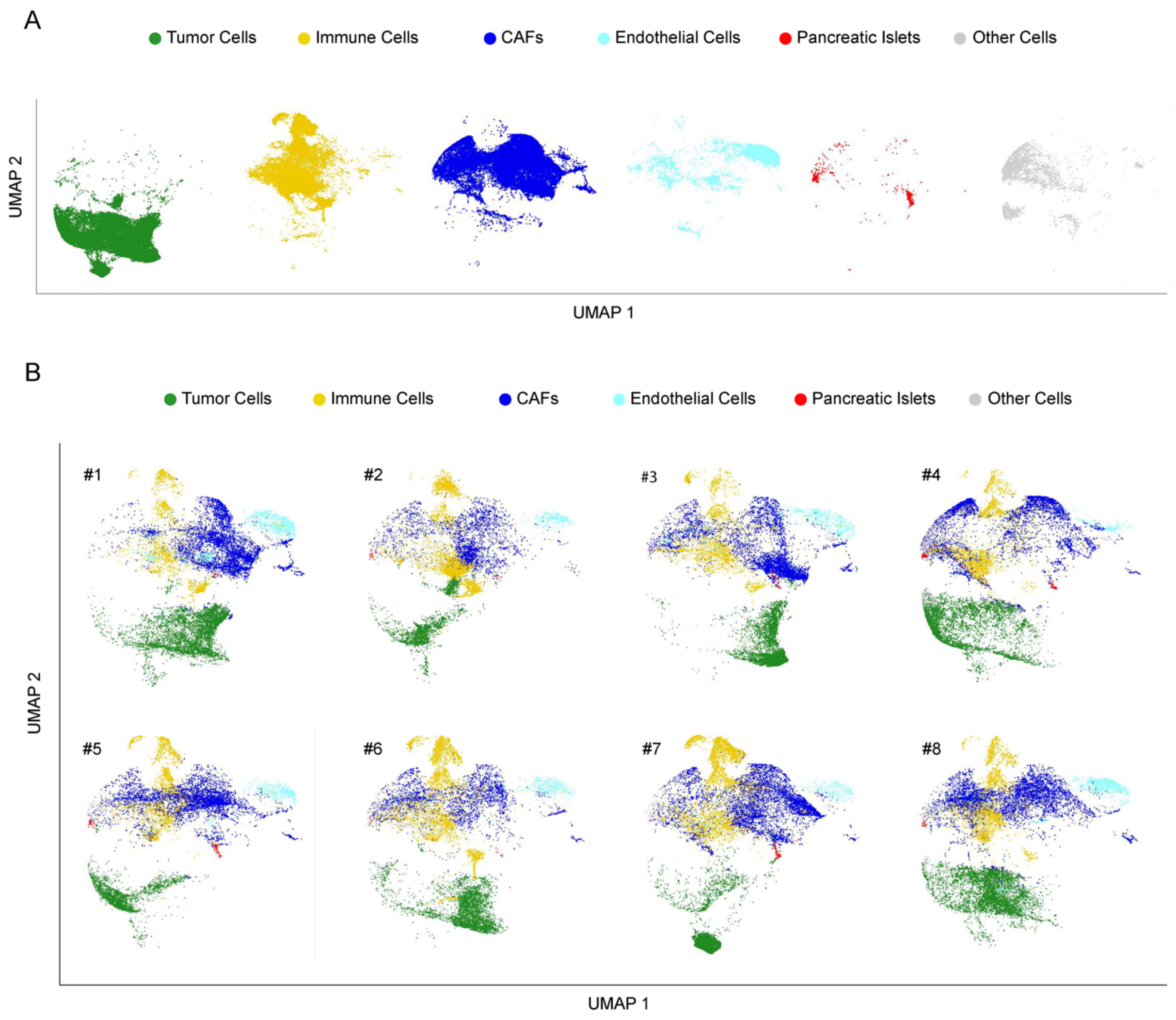

Supplementary Figure 2: UMAP representation of PDAC annotated cells. A) Separated UMAP representation, over all the acquired images, of PDAC cells annotated into tumor cells, immune cells, CAFs, endothelial cells, pancreatic islet and other cells, as in the legend. **B**) UMAP representation, over all the acquired images, of PDAC cells annotated into tumor cells, immune cells, CAFs, endothelial cells, pancreatic islet and other cells in each PDAC of the analysed cohort, as in legend.

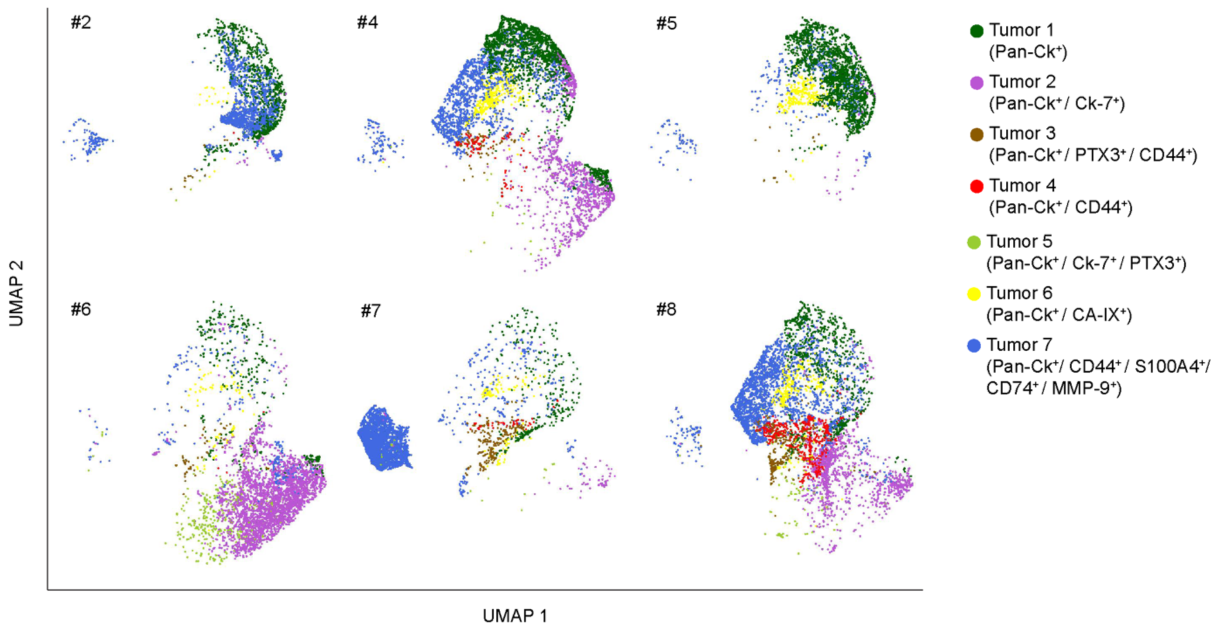

Supplementary Figure 3: UMAP representation of PDAC tumor cells per patient. UMAP representation, over all the acquired images, of PDAC tumor cell subtypes in each patient of the analysed cohort, as in legend.

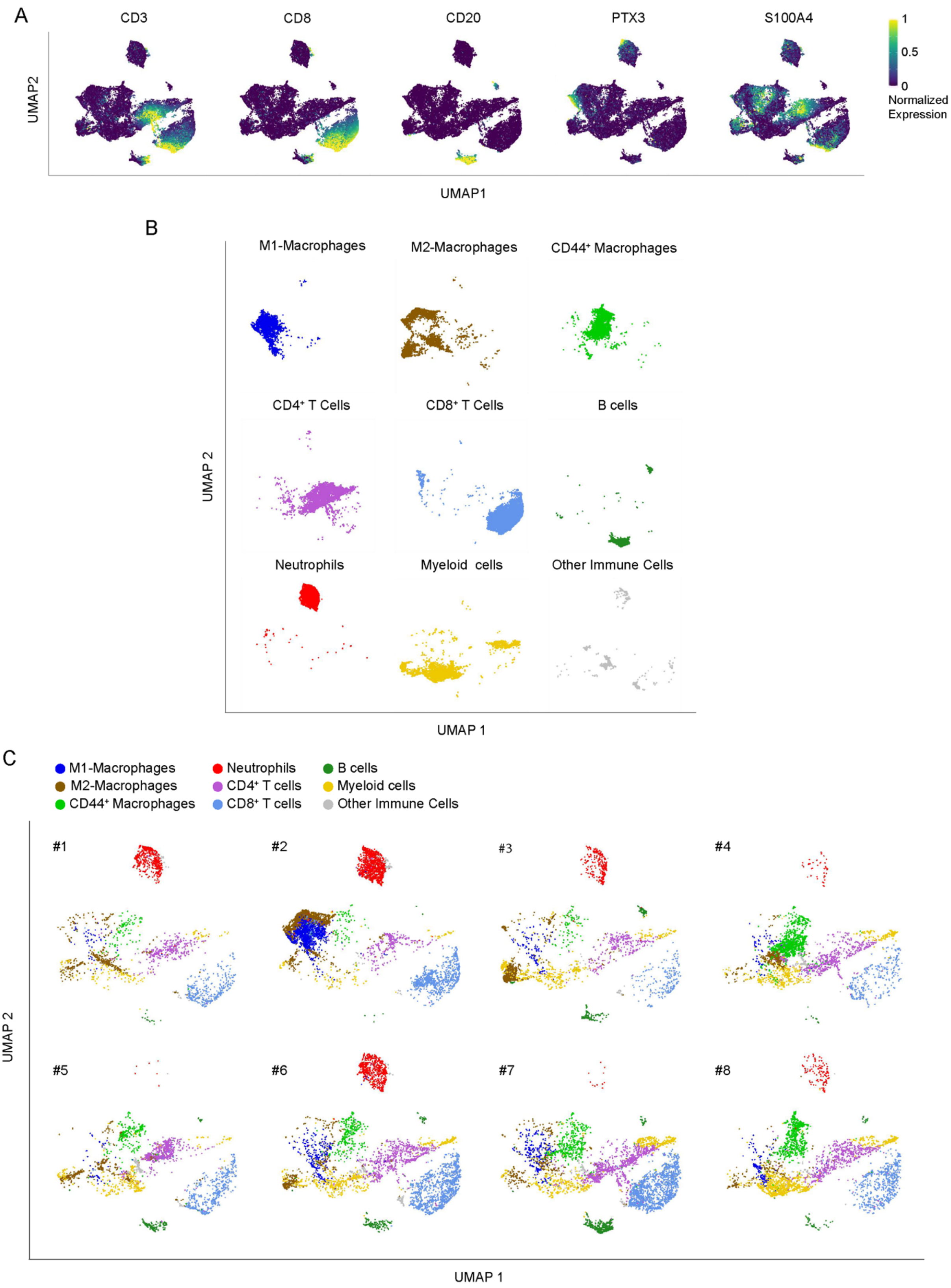

Supplementary Figure 4: UMAP representation of PDAC immune cells. A) UMAP representation of the normalized expression of immune cell markers for the identification of T cells (CD3 and CD8), B cells (CD20) and for the expression of functional markers PTX3 and

S100A4. **B**) Separated UMAP representation, over all the acquired images, of PDAC immune cells annotated into M1-Macrophages, M2-Macrophages, $CD44^{+}$ Macrophages, $CD4^{+}$ T cells, $CD8^{+}$ T cells, B cells, Neutrophils, Myeloid cells and other immune cells. **C**) UMAP representation, over all the acquired images, of PDAC immune cells annotated into M1-Macrophages, M2-Macrophages, $CD44^{+}$ Macrophages, $CD4^{+}$ T cells, $CD8^{+}$ T cells, B cells, Neutrophils, Myeloid cells and other immune cells in each patient of the analysed cohort, as in legend.

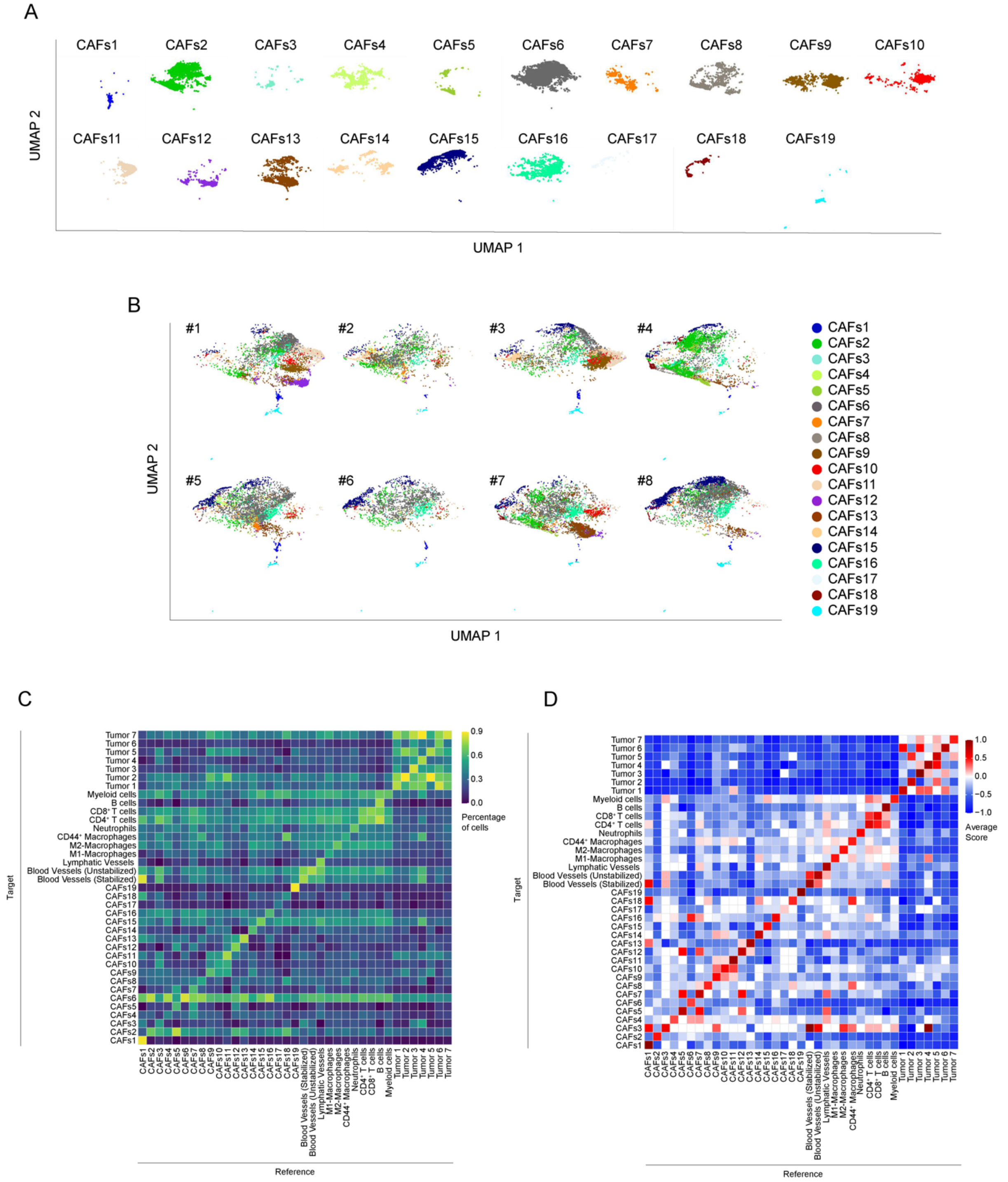

Supplementary Figure 5: UMAP representation of PDAC CAFs. A) Separated UMAP representation, over all the acquired images, of PDAC CAFs subtypes. **B**) UMAP representation, over all the acquired images, of PDAC CAF subtypes in each patients of the analysed cohort, as in legend. **C**) Results of the neighbourhood analysis, as heatmap, showing the average percentage of each indicated cell subtype (Reference) that are in proximity (≤ 30μm radius) to each indicated cell subpopulations (Target). **D**) Results of the neighbourhood analysis, as heatmap, showing the average proximity score for each pair of cell phenotypic

subpopulations of CAF subtypes (Reference) to immune cells (Target). Positive (red) or negative (blue) values indicate that a specific pair of phenotypes is neighbouring significantly more often or significantly less often, respectively, than expected from a randomized placement, as described in Material and Methods. 30μm radius is considered for cell-to-cell proximity.

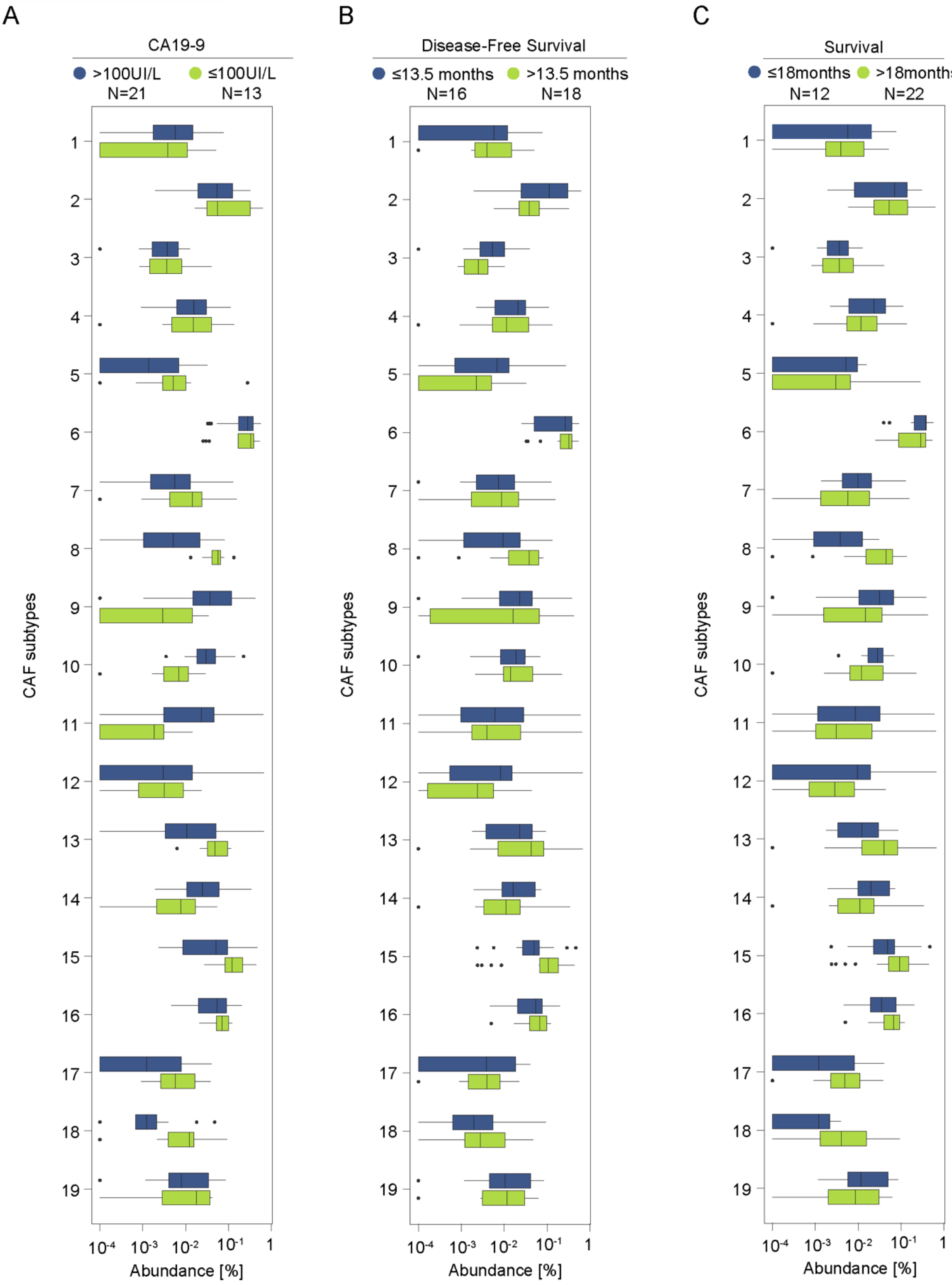

Supplementary Figure 6. Association between CAFs and patients' levels of CA19-9, disease-free survival (DFS) and survival status. A) Box plot showing relative abundance of CAF subtypes in each ROI (34 acquired ROIs, from n=8 PDAC) grouped according to patients' clinical parameters: pre-operative levels of CA19-9 (blue >100IU/l, green ≤ 100IU/l), disease-free survival (DSF, blue ≤13.5 months, green >13.5 months) and survival (blue ≤18 months, green >18 months) after the surgery.
